# Supplementary material for: Evolving cooperation in multichannel games
Source: Nat Commun. 2020 Aug 4;11:3885. doi: 10.1038/s41467-020-17730-3 (PMC7403149; doi:10.1038/s41467-020-17730-3)
Supplement: Supplementary file 1 — Supplementary Information [file 41467_2020_17730_MOESM1_ESM.pdf]

Supplementary Information

Evolving cooperation in multichannel games

Donahue et al.

Contents

Supplementary Figures **2**

Supplementary Note 1: Related literature and novelty **12**

Supplementary Note 2: Model description **14**
Game setup . . . 14
Reactive strategies . . . 15
Calculation of payoffs . . . 17
Evolutionary dynamics . . . 20

Supplementary Note 3: Equilibrium analysis **21**
Partners, semi-partners, and defectors . . . 21
Characterization of Nash equilibria in multichannel donation games . . . 22
Relative abundance of partners, semi-partners and defectors . . . 26
Numerical identification of strategies . . . 27

Supplementary Note 4: Model extensions **28**
Memory-1 strategies . . . 28
Different types of games . . . 31

Supplementary Note 5: Appendix **33**
Proofs of the equilibrium results . . . 33
MATLAB code used for the numerical results . . . 41

Supplementary References **44**

Supplementary Figures

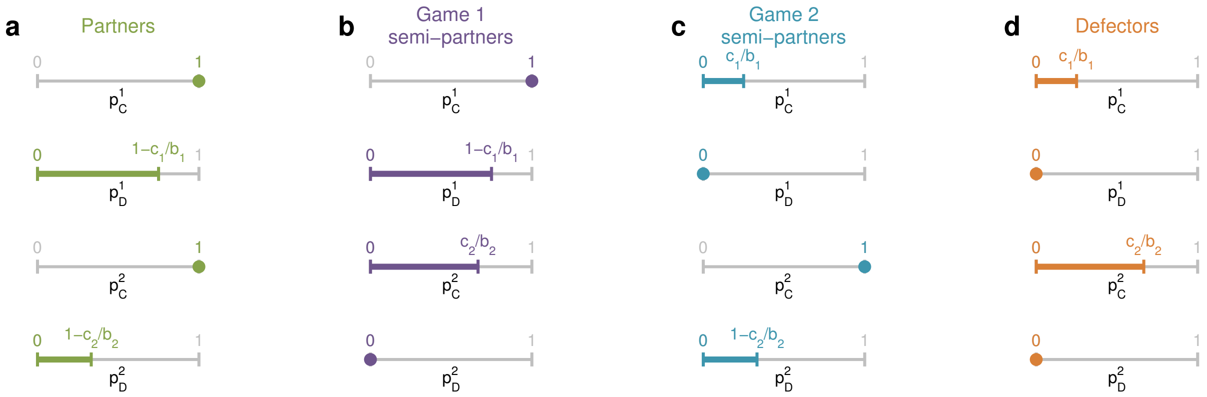

Supplementary Figure 1: Stable reactive strategies in the unlinked case. We can characterize the sets of partners, semi-partners, and defectors among the reactive strategies analytically, for an arbitrary number m of games. Here we depict the respective conditions for $m=2$. **a,** Partners always respond to a co-player's cooperation in a given game by cooperating as well (represented by a green dot). If the co-player defected, the partner's cooperation probability needs to be bounded by $1-c_k/b_k$ for each game k (represented by a green interval). In particular, *ALLC* is not a partner strategy. The characterizations of semi-partners (**b,c**) and defectors (**d**) is similar. Here we depict the case of two prisoner's dilemma games with $b_1=4$, $b_2=3/2$, and $c_1=c_2=1$.

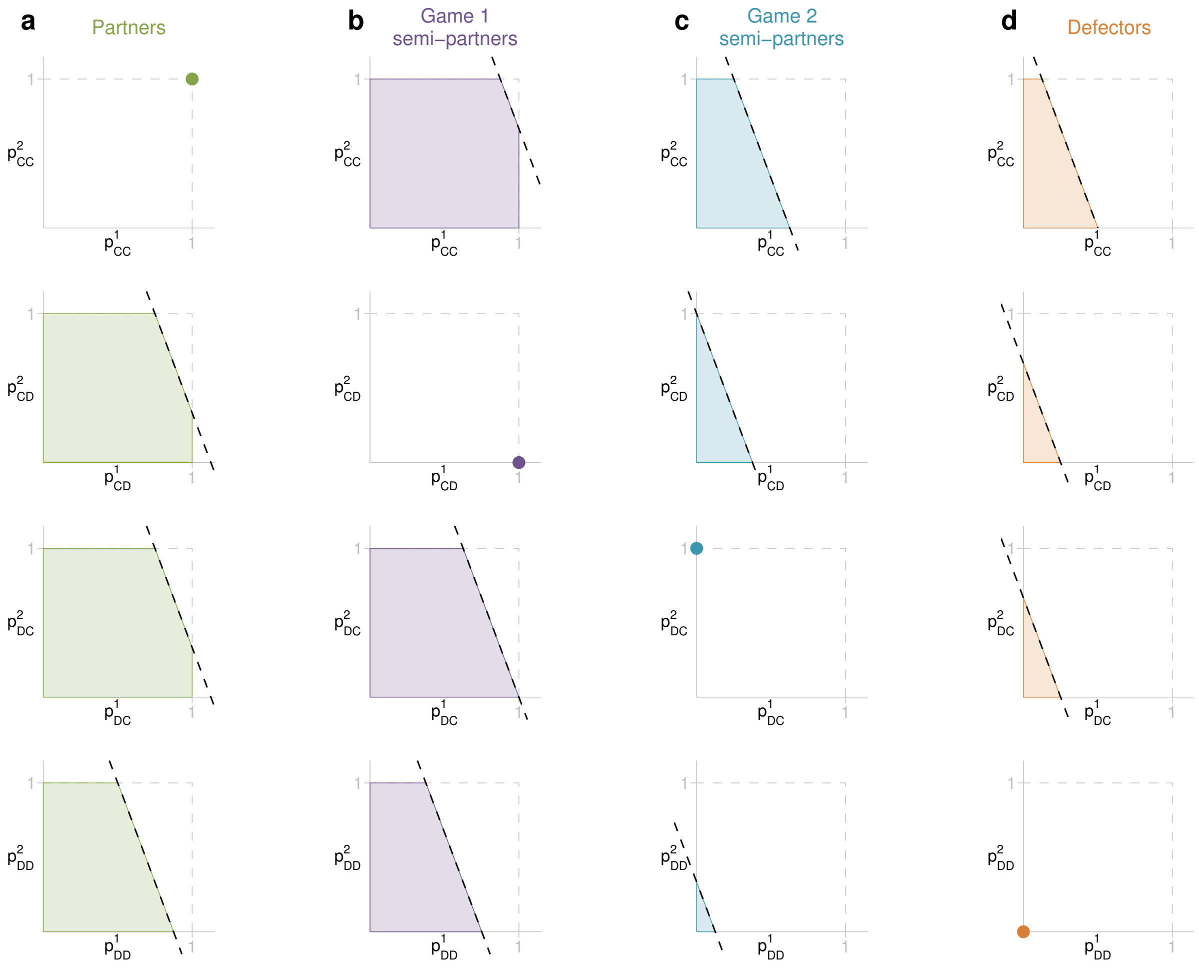

Supplementary Figure 2: Stable reactive strategies in the linked case. When games are linked, players can flexibly respond to a co-player's transgression by reducing their cooperation probability in either game. Here, we depict the resulting equilibrium conditions for partners (**a**), semi-partners (**b,c**), and defectors (**d**) for each of the possible co-player's behaviors in the previous round. These possible behaviors are CC (co-player cooperated in both games), CD or DC (co-player either only cooperated in the first or in the second game), and DD (co-player defected in both games). The respective equilibrium conditions are represented by colored dots and areas (for the respective algebraic expressions, see **SI**). After each deviation of the co-player, players are required to defect in at least one of the games with positive probability. Parameters are the same as in **Supplementary Fig. 1**.

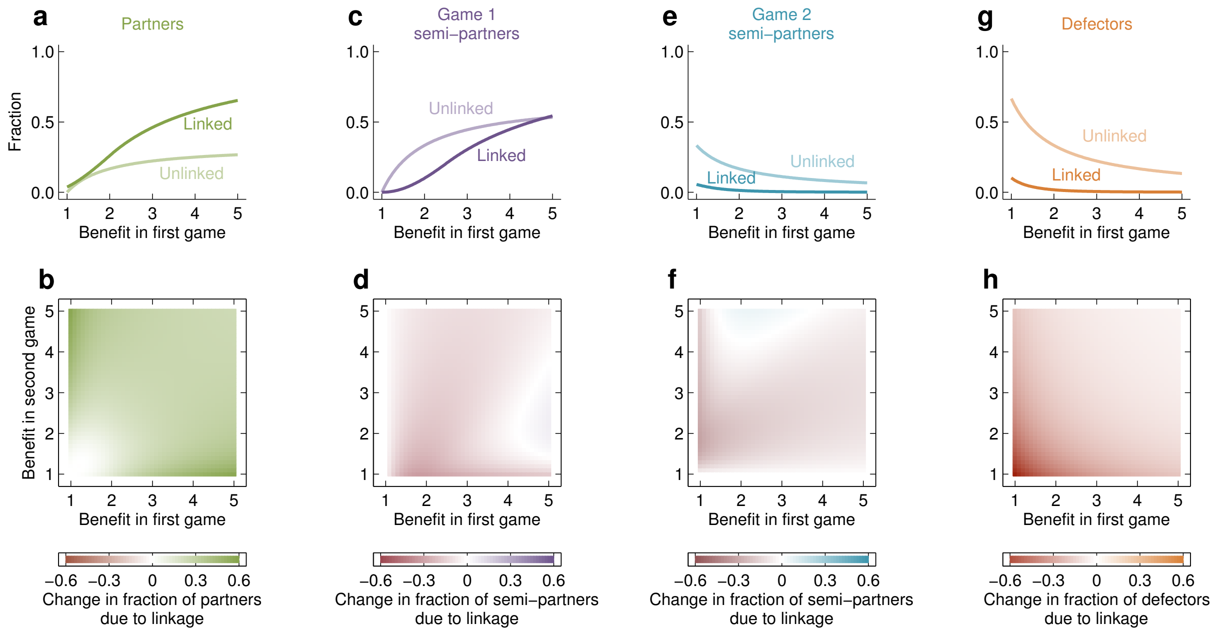

Supplementary Figure 3: When players are able to link different games, partners become more abundant. **a,** We can analytically calculate the fraction of partners among those strategies that fully cooperate against each other in both games. This fraction is a measure for how likely individuals are to find a strategy in the vicinity of partners when they randomly explore new strategies through mutations. The precise formulas are in the **SI**. We find that partners have a higher relative abundance when the two games are linked. **b,** To explore how the relative abundance of partners depends on parameters, we simultaneously vary the benefit of cooperation in the two games. The color depicts the difference between the fraction of partners in the linked case and the respective fraction in the unlinked case. According to the graph, the linked case is particularly beneficial compared to the unlinked case when the benefit of cooperation is large in one game and small in the other. But even if the two games provide similar benefits of cooperation, linkage tends to be advantageous. **c–h,** Similarly, we can also compute the relative abundance of semi-partners and defectors. For most of the parameter space, these strategy classes become less abundant when the two games are linked. For the top panels, we vary b_1 and keep $b_2 = 3/2$ and $c_1 = c_2 = 1$. For the bottom panels, we additionally vary b_2.

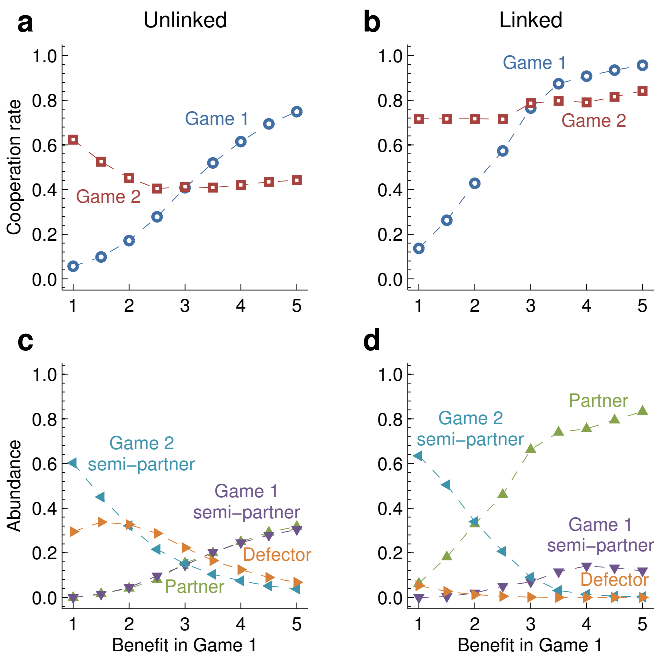

Supplementary Figure 4: The advantages of linkage across different benefit-to-cost ratios. a, To show that the positive effect of linking is persistent, we have run further simulations in which we systematically vary the benefit of cooperation in the first game. Across all parameter values, we find higher cooperation rates in both games when the games are linked. Interestingly, in the unlinked case, increasing the benefit of cooperation in the first game leads to a reduced cooperation rate in the second. This is a hitchhiking effect: When b_1 is comparably large, strategies are mainly selected for how well they perform in the first game, whereas their performance in the second game becomes increasingly irrelevant (see also **Supplementary Fig. 5**). **b,** We have computed how often players adopt one of the four strategy classes of partners, game 1 semi-partners, game 2 semi-partners, and defectors. Partner strategies are more frequently played in the linked case, which explains the prevalence of cooperation in this scenario. Parameters are the same as in **Fig. 3**.

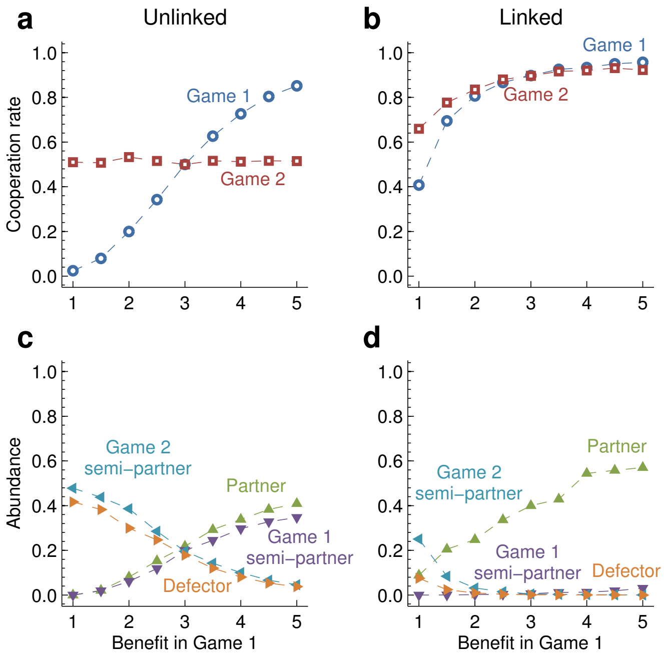

Supplementary Figure 5: Evolution of cooperation under independent mutations. For our previous simulations we have assumed that mutations affect a player's behavior in both games simultaneously. Here, we present further simulations in which mutations occur independently. We use the same basic setup as in **Supplementary Fig. 4**, but this time mutations only affect a player's behavior in one of the games (the mutation is equally likely to occur in game 1 and game 2). While linkage again increases cooperation in both games, there are two crucial differences compared to **Supplementary Fig. 4**. First, in the unlinked case, the cooperation rate in game 2 is now independent of the benefit of cooperation in game 1, as expected. Second, in the linked case, the highlighted strategy classes of partners, semi-partners, and defectors now explain a smaller fraction of the evolving strategies. In particular, for $b_1 = 5$, the fraction of partners is below 60%, although most players cooperate in both games. This effect occurs because under this mutation scenario strategies can be evolutionarily robust even if they are not Nash equilibria. As an example, we frequently observe strategies that cooperate if and only if the co-player either cooperated in both games or defected in both games, $\mathbf{p} = (1, 0, 0, 1;\ 1, 0, 0, 1)$. This strategy is not a Nash equilibrium because *ALLD* can invade. However, the strategy is robust under independent mutations, because a player who starts defecting only in one game is punished in both. Parameters are the same as in **Supplementary Fig. 4**.

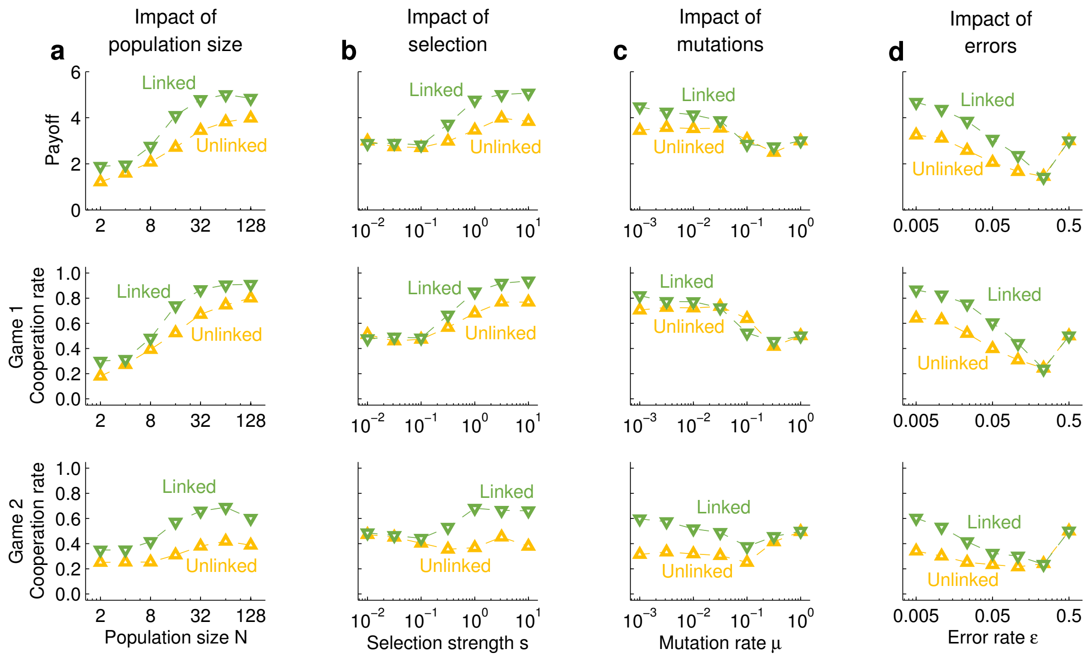

Supplementary Figure 6: Robustness with respect to changes in the evolutionary parameters. For all previous simulations on evolution among reactive players, we have used as fixed parameters a population size of $N = 50$, selection strength $s = 2$, mutation rate $\mu \to 0$, and error rate $\varepsilon = 0$. Here we show that we obtain similar results for reactive players when we vary these parameters, provided that (**a**) the population is sufficiently large, (**b**) selection is strong, (**c**) mutations are sufficiently rare, and (**d**) errors are not too abundant. For this figure, we use the same setup and the same baseline parameters as in **Fig. 2**, and we only vary the parameters explicitly mentioned. Each symbol represents the corresponding final value after 60,000 time steps of a simulation, averaged over 300 simulations.

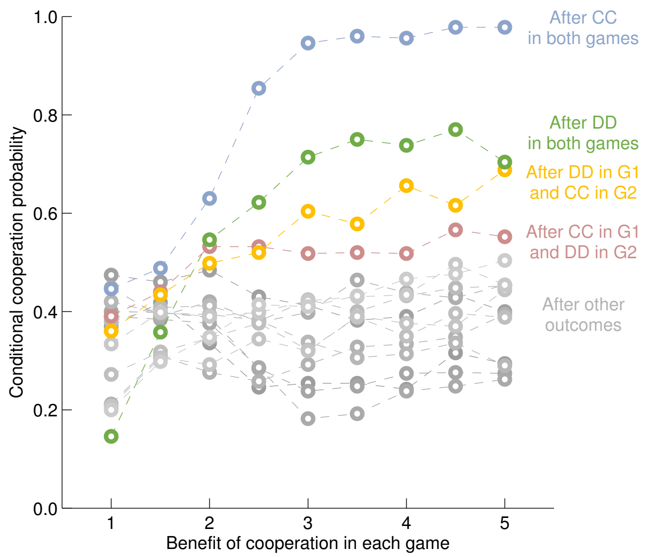

Supplementary Figure 7: Memory-1 players learn to adopt *CIC* when games are linked. To explore the persistence of *CIC*-like strategies, we have repeated the simulations in **Fig. 4** while varying the benefit of cooperation b, which we assume to be identical in both games. If *CIC* is favored by selection, we would expect that players have the highest cooperation probability after rounds in which their actions coincided in each game (either CC or DD). The graph suggests that behavior consistent with *CIC* evolves for $b > 2c$, that is when *CIC* is an equilibrium (see **SI**). Except for the benefit values, we use the same parameters as in **Fig. 4**, and we average over 500 simulations for each data point. Because here we consider the case that players engage in two identical games, we only show the cooperation probabilities for the first game.

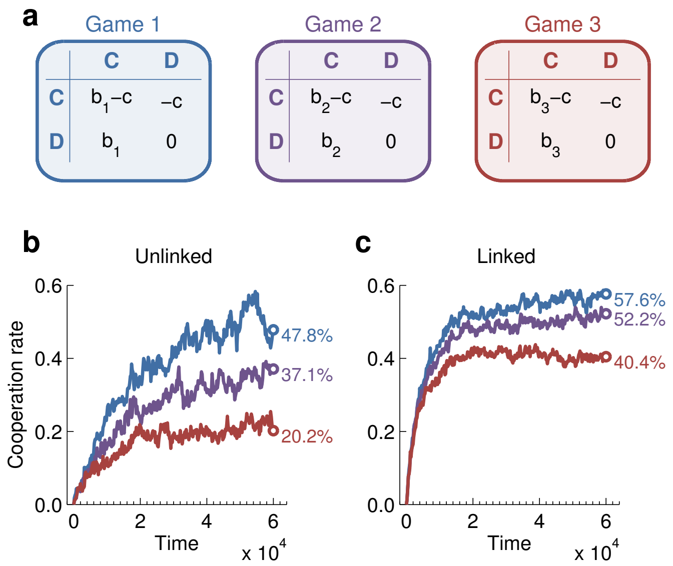

Supplementary Figure 8: The benefits of linkage in a multichannel game with three games. We have run simulations for a scenario in which players interact across three different prisoner's dilemmas. The different games are ordered according to their benefit of cooperation, with $b_1 > b_2 > b_3$. Again, linkage enhances cooperation in each game. For the payoff parameters, we use $b_1 = 4$, $b_2 = 3$, $b_3 = 2$, and $c_k = 1$ for all games k. All other parameters and the general setup is the same as in **Fig. 2**. The data represents averages over 150 independent simulations.

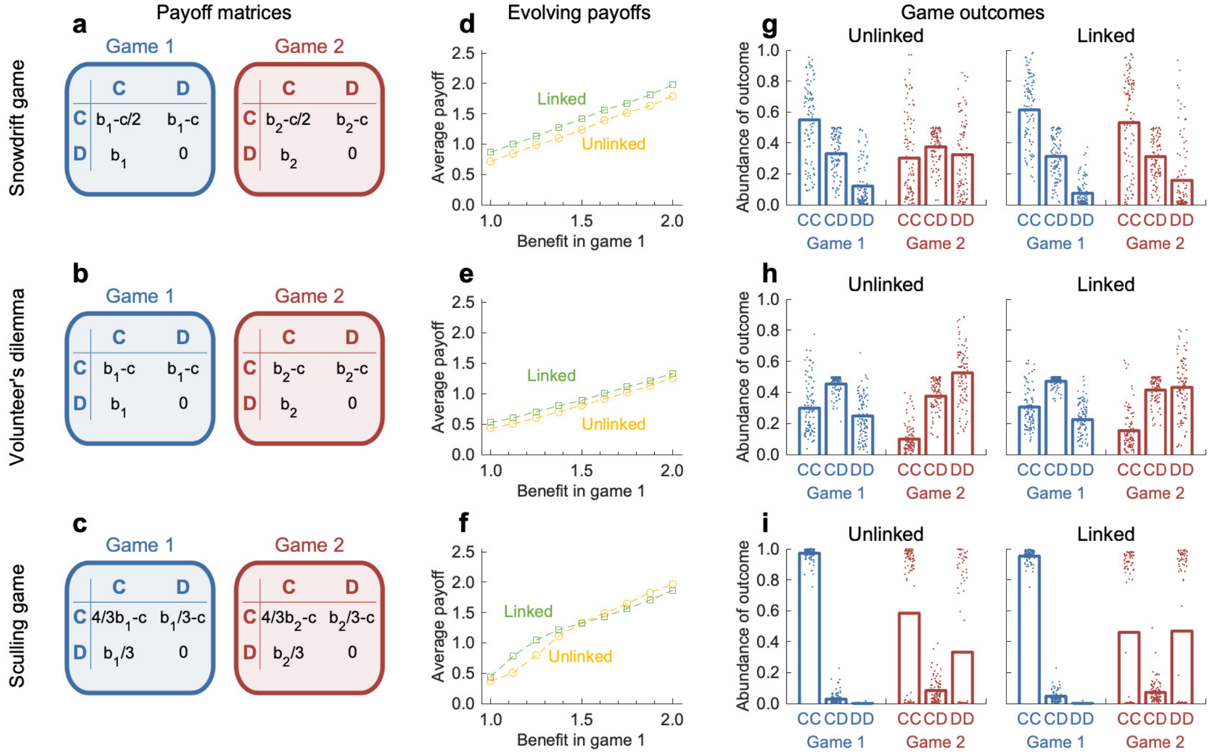

Supplementary Figure 9: The effect of linkage in the snowdrift game, the volunteer's dilemma, and the sculling game. a, In the snowdrift game[69,70], at least one of the two players needs to cooperate for both players to derive a benefit b. If both players cooperate, they share the cost. It follows that mutual defection is no longer an equilibrium of the one-shot game. The symmetric social optimum is achieved when players either mutually cooperate in each round, or if they cooperate in an alternating fashion. **b,** The volunteer's dilemma[71] is similar to the snowdrift game, but cooperators pay the full cost in any case. Again mutual defection is not an equilibrium of the one-shot game. However, the symmetric social optimum requires (anti-)coordination; players need to cooperate in turns. **c,** In the sculling game[72], the benefit of cooperation is nonlinear in the number of cooperators. For the chosen parameters, this gives rise to a coordination game. In particular, even if the game is only played once, mutual cooperation is an equilibrium. **d–f,** For the first two game types, simulations show that players yield larger average payoffs in the linked case. For the sculling game, this relationship can reverse for large benefits of cooperation (when cooperation becomes risk-dominant). **g,** For the snowdrift game, linking increases the likelihood that the two players cooperate in both games. **h,** For the volunteer's dilemma, linking leads to fewer defections, but players do not necessarily coordinate on an equilibrium in which players alternate to volunteer. **i,** If the benefit of cooperation is sufficiently large (here for game 1), players are fully cooperative in both the linked and the unlinked case. However, linkage can still affect how likely players coordinate on cooperation in the less profitable game 2. For the game parameters in panels **d–f**, we used $c = 1$ and $b_2 = 1.2$ throughout. The evolutionary parameters are the same as in **Fig. 2**. We have run 500 simulations with 10^5 time steps for each data point. The bar diagrams in panels **g–i** shows the abundance of each possible outcome when we additionally set $b_1 = 2$. Here, each bar shows the respective mean over all simulations, whereas dots represent individual outcomes for 100 sample simulations.

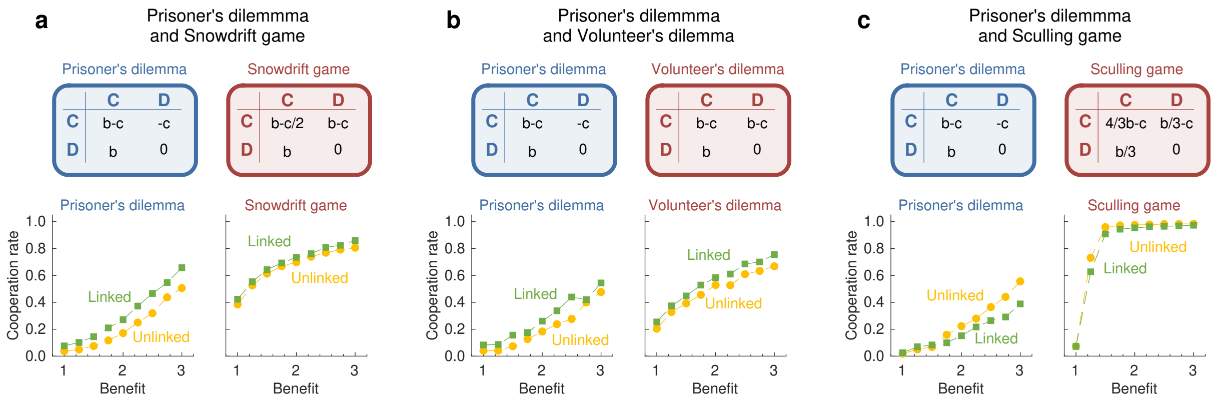

Supplementary Figure 10: The effect of linkage when different game types are combined. The framework of multichannel games does not require that players interact across several games of the same type. Instead, here we show simulation results for the case that one of the games is a prisoner's dilemma whereas the other one is either a snowdrift game (**a**), a volunteer's dilemma (**b**), or a sculling game (**c**). We observe in the first two cases that players are more cooperative when games are linked. Only for the sculling game, we again observe that linkage can result in lower cooperation rates. This effect is particularly pronounced for large benefits of cooperation. For the simulations shown here we assumed that the benefit parameter is the same in both games, keeping $c=1$ fixed. The evolutionary parameters are the same as in **Supplementary Fig. 9**. Again, all data points show averages over 500 simulations, with each simulation being run for 10^5 time steps.

Supplementary Note 1: Related literature and novelty

Herein, we explore how individuals cooperate when they simultaneously interact in several concurrently ongoing games with the same co-player. In the following, we describe the current status of the respective literature. In particular, we discuss previous attempts to study joint behavior in multiple games and we highlight what our study adds to this literature.

Most relevant to our paper is previous work on evolutionary multi-game dynamics[1–4]. This literature assumes that players are engaged in several distinct one-shot games. In particular, players do not use their experiences from previous interactions to adapt their future behavior. In an early paper on the subject, Cressman *et al*[1] consider the case that two individuals interact in a multi-game consisting of two different one-shot games. They show that the Nash equilibria and the evolutionarily stable strategies of the multi-game can be directly inferred from the one-shot games. In a companion paper, however, Chamberland and Cressman[2] prove that multi-games differ from the individual one-shot games with respect to their dynamical properties. To this end, they discuss an example where the replicator dynamics of each one-shot game converges to a unique equilibrium, whereas the multi-game yields persistent cycles. Subsequent work has observed similar dynamical inconsistencies when there are more than two one-shot games, arbitrarily many strategies, and more than two players[3,4]. Our approach differs from this literature in the following key aspects:

(*i*) We consider repeated interactions. In particular, we explore which behaviors emerge if players can use information from previous encounters in one game to modify their actions in another unrelated game.

(*ii*) We characterize which strategies stabilize cooperation when each game is a social dilemma. This question cannot be explored within a one-shot framework, which would predict mutual defection in all games.

(*iii*) We observe substantial effects even in simple games with two players and two games with two actions each. In a one-shot setting there need to be at least three actions in each game for any inconsistencies to occur[1].

In addition, several papers discuss which equilibria can arise when firms compete in several distinct markets[5–7]. For example, in an influential early study on the subject, Bernheim and Whinston analyze a series of models in which two firms repeatedly engage in price competition across multiple markets. Their results are two-fold.

First, they show an irrelevance result: When markets and firms are identical, and when the production technology exhibits linear returns of scale, multimarket contact does not enhance the firms' abilities to sustain a collusive outcome. Intuitively, when the discount factor (the constant rate with which future payoffs are discounted) is too small, collusion is impossible even for multimarket competition. On the other hand, once the discount factor is sufficiently large, firms can reach a collusive agreement even without multimarket contact.

Second, they show that each assumption of the irrelevance result is binding. If either markets or firms differ, or if there are nonlinear production costs, multimarket contact may facilitate collusive agreements. Moreover, multimarket contact can promote collusion even under fully symmetric conditions if individual actions can only be monitored imperfectly[6]. We add to this literature as follows.

(*i*) Previous models of multimarket contact take a static perspective. They explore whether multimarket contact changes the set of outcomes that can be sustained in equilibrium. Our study adds an evolutionary perspective. We explore how populations choose between different equilibria, depending on whether or not players link the different games they are engaged in.

(*ii*) To show whether multimarket contact enhances cooperation among firms, it is sufficient to construct *one* equilibrium strategy with the desired properties. In contrast, for our evolutionary approach it is crucial to know how rich the corresponding set of strategies is. In **Supplementary Note 3**, we thus characterize *all* equilibrium strategies within the space of reactive strategies that give rise to a given outcome.

(*iii*) We study the case of repeated games without discounting. For that case, previous models would not predict any effect at all, because each individual game on its own already allows for full cooperation. In contrast, we find that linking increases the relative abundance of partner strategies, and thereby the chance that individuals will settle on a fully cooperative equilibrium.

Somewhat more remote to our paper are studies in which a social dilemma is coupled with a second game in which individuals can either punish or reward each other[8–24]. Here, the second game does not entail any cooperation motive by itself. Instead, it is explicitly introduced to promote cooperation in the first game. The extensive experimental evidence on such combined games suggests that punishment can increase contributions to public goods[8]. However, due to the punishment costs, overall welfare may be reduced, especially if the game is only played for a few rounds[9–12]. In contrast, games in which individuals can reward cooperators seem to increase contributions without any negative repercussions[14,15]. Importantly, the respective results are explained through models of one-shot interactions[16–24]. While the incentives provided in the second game can easily explain the evolution of cooperation in the primary game, the incentives themselves require external mechanisms to evolve. The additional mechanisms considered include reputation formation[16], optional participation[17], spatial structure[19,20], central institutions[22,23], or linkage between first- and second-order punishment[24]. We add to this literature as follows.

(*i*) We show that individuals learn to coordinate their play across independently ongoing social dilemmas to mutually stabilize cooperation in each of them.

(*ii*) Our approach does not require any external institutions to incentivize cooperation, nor does it evoke additional mechanisms such as spatial structure or group selection.

(*iii*) Previous work on endogenous sanctioning suggests that punishment is sometimes misdirected at either those who cooperate[10] or those who punish[25]. Theoretical models indicate that instances of anti-social rewarding may also occur[26,27]. In contrast, within our model such antisocial behaviors are selected against, and they do not arise in any equilibrium.

Supplementary Note 2: Model description

Game setup

In the following we describe our framework in full detail. We consider multichannel games between two players. The players interact in m different (elementary) games. In each such game, players can either cooperate (C) or defect (D). The payoffs for each game depend on the players' actions in the respective game, but they are independent of the actions in the other $m-1$ games. Payoffs are symmetric. Thus the outcome of each game k can be represented by a 2×2 payoff matrix

$$\begin{array}{c} \\ C \\ D \end{array} \begin{array}{c} \begin{array}{cc} C & D \end{array} \\ \left(\begin{array}{cc} R_k & S_k \\ T_k & P_k \end{array}\right) \end{array}. \tag{1}$$

Here, R_k is the reward a cooperator obtains against another cooperator; S_k is the sucker's payoff that a cooperator obtains against a defector; T_k is the temptation payoff a defector obtains against a cooperator; and P_k is the punishment payoff for mutual defection[28]. Without loss of generality, mutual cooperation is preferred over mutual defection, such that $R_k > P_k$. The prisoner's dilemma satisfies the additional conditions $T_k > R_k$ and $P_k > S_k$, which results in the most stringent case of a social dilemma[29,30].

The m games are played repeatedly and in parallel. Players interact for infinitely many rounds. In each round they decide independently for each game whether or not to cooperate, without knowing the respective choices of the co-player. After each round, players learn their co-player's actions in all m games, and which payoffs the two players receive as a result. If $\pi_i^k(t)$ is the resulting payoff player i receives in game k in round t, we define the player's repeated game payoff in game k as the average

$$\pi_i^k = \lim_{\tau\to\infty} \frac{1}{\tau} \sum_{t=1}^{\tau} \pi_i^k(t). \tag{2}$$

Because we study players with finite memory, the existence of the limit in Eq. (2) is guaranteed, and payoffs can be calculated explicitly (see Section "Calculation of payoffs" for details). We define the player's payoff in the multichannel game as the sum over all payoffs in the m repeated games,

$$\pi_i = \pi_i^1 + \ldots + \pi_i^m. \tag{3}$$

Player thus aim to maximize their payoff across all games and rounds.

The players' actions may be subject to noise. Each time a player decides to cooperate or defect in a given game, there is a uniform probability ε that the player implements the opposite action. In addition to making the model more realistic, this inclusion of errors comes with two theoretical advantages.

First, errors make the players' payoffs independent of initial play[31]. Herein, we consider infinitely repeated games without discounting among players with finite memory. For such games, errors ensure

that the players' first round behavior has no lasting impact; eventually, the players' behavior is independent of what happens in the beginning of the game. This observation reduces the dimensionality of the strategy space. Strategies do not need to specify what to do in the first round.

Second, previous work on repeated games has shown that no strategy is evolutionarily stable in the absence of errors[32–34]. Intuitively, if all players follow the same (deterministic) strategy, they never experience certain game histories. As a consequence, there is no selection against mutant strategies that only deviate after histories that do not occur in equilibrium. Once neutral mutants reach a sizable proportion of the population, they may in turn favor the selective invasion of further mutant strategies. In contrast, in games with errors such neutral invasions are ruled out, as any finite game history is observed with positive probability. As a result, evolutionarily stable cooperation becomes feasible[35].

To explore how subjects learn to coordinate their play across the m games, we consider two different scenarios. We refer to these scenarios as the *unlinked case* and the *linked case*.

The unlinked case serves as the control scenario. Here, any strategic attempt to use cooperation in one game as leverage to increase cooperation in another game is ruled out by assumption. Players are only permitted to use strategies in which their behavior in game k is independent of what happened in all other games. Such a scenario could occur for example, if each game is played by a different subunit of the player (for example, a firm), and subunits are not informed about the other games (or the subunits are not incentivized to take the other games into account).

In the linked case, players may react to a co-player's action in one game by changing their action in all m games. We note that the linked case allows for all behaviors that the unlinked case allows for: a player may simply choose to treat all her different games as independent. We evaluate the strategic advantage of linking the different games by (*i*) comparing the evolving cooperation rates of the two cases, and by (*ii*) quantifying how often players use strategies in the linked case that are infeasible in the unlinked case.

Reactive strategies

Strategies in a repeated game are contingent rules that tell the player what to do in any given round, depending on the outcome of all previous rounds[36]. Because any non-generic repeated game allows for infinitely many strategies, the same is true for multichannel games, in which m repeated games are combined. To make a computational analysis of the evolutionary dynamics feasible, it is thus useful to restrict the players' strategy space by only considering strategies of some given complexity[37].

For the baseline model, we have used the space of reactive strategies. Such strategies only depend on the co-player's behavior in the previous round. To introduce these strategies formally, suppose the co-player's previous action in game k is $a_k \in \{C, D\}$. Let $\mathbf{a} = (a_1, \ldots, a_m)$ denote the corresponding action profile. A reactive strategy then needs to specify with which probability $p^k_{\mathbf{a}}$ the focal player is to cooperate in the next round, for each game k. The space of reactive strategies for the linked case is

therefore given by

$$\mathcal{R}_L = \left\{ \mathbf{p} = (p^k_{\mathbf{a}})_{\mathbf{a}\in\{C,D\}^m,\ k\in\{1,\dots,m\}} \;\middle|\; p^k_{\mathbf{a}} \in [0,1] \text{ for all } k \text{ and } \mathbf{a} \right\}. \tag{4}$$

When there are m games, there are 2^m possible action profiles $\mathbf{a}$, and hence $\mathcal{R}_L$ corresponds to the $m\cdot 2^m$-dimensional unit cube.

In the unlinked case, players are unable to condition their game k behavior on the outcome of the other games. One way to introduce unlinked strategies is thus to define them as the subspace of $\mathcal{R}_L$ where a player's response in game k is required to be independent of the co-player's action in the other games,

$$\tilde{\mathcal{R}}_U := \left\{ \mathbf{p} = (p^k_{\mathbf{a}}) \in \mathcal{R}_L \;\middle|\; p^k_{\mathbf{a}} = p^k_{\tilde{\mathbf{a}}} \text{ for all action profiles } \mathbf{a} \text{ and } \tilde{\mathbf{a}} \text{ for which } a_k = \tilde{a}_k \right\}. \tag{5}$$

However, to simplify notation it is useful to define the space of reactive strategies for the unlinked case as the elements of

$$\mathcal{R}_U = \left\{ \mathbf{p} = (p^1_{a_1};\ p^2_{a_2};\ \dots\ ;\ p^m_{a_m})_{a_k\in\{C,D\},\ k\in\{1,\dots,m\}} \;\middle|\; p^k_{a_k} \in [0,1] \text{ for all } k \right\}. \tag{6}$$

Here, $p^k_{a_k}$ is the player's conditional cooperation probability in game k, depending on the co-player's previous action a_k in that game. The two definitions for reactive strategies for the unlinked case are equivalent: Each strategy $\mathbf{p} \in \mathcal{R}_U$ can be uniquely mapped to a strategy $\tilde{\mathbf{p}} \in \tilde{\mathcal{R}}_U$ by defining $\tilde{p}^k_{(a_1,\dots,a_m)} = p^k_{a_k}$. From representation (6) it follows that the space of reactive strategies in the unlinked case is $2m$-dimensional.

Let us illustrate these definitions with two examples.

1. When there is only one game, the space of reactive strategies for the linked and the unlinked case coincide. In both cases, reactive strategies take the conventional format $\mathbf{p} = (p^1_C, p^1_D)$ familiar from the previous evolutionary literature on repeated games[38,39]. Here, p^1_a is a player's cooperation probability depending on whether the co-player cooperated (a = C) or defected (a = D) in the previous round. Using this notation, the strategy ALLD corresponds to the vector (0,0), ALLC to (1,1), and Tit-for-Tat (TFT) to (1,0).

2. When there are two games (as considered in the main text), reactive strategies in the unlinked case take the form

$$\mathbf{p} = (p^1_C, p^1_D;\ p^2_C, p^2_D). \tag{7}$$

   The value of p^k_a is the player's cooperation probability in game k, depending on whether the co-player previously cooperated (a = C) or defected (a = D) in that very game. In contrast, reactive

strategies in the linked case are given by

$$\mathbf{p} = (p^1_{CC}, p^1_{CD}, p^1_{DC}, p^1_{DD};\; p^2_{CC}, p^2_{CD}, p^2_{DC}, p^2_{DD}). \tag{8}$$

Here, $p^k_{(a_1,a_2)}$ is the probability to cooperate in game k, conditional on the co-player's previous actions in both games. An unlinked strategy $\mathbf{p} = (p^1_C, p^1_D;\; p^2_C, p^2_D) \in \mathcal{R}_U$ can be implemented within the linked strategy space $\mathcal{R}_L$ by defining

$$\begin{aligned}
p^1_{CC} &= p^1_{CD} := p^1_C \\
p^1_{DC} &= p^1_{DD} := p^1_D \\
p^2_{CC} &= p^2_{DC} := p^2_C \\
p^2_{CD} &= p^2_{DD} := p^2_D.
\end{aligned} \tag{9}$$

In particular, if we are to implement the game-wise TFT strategy, the unlinked version (1,0; 1,0) translates into the strategy (1,1,0,0; 1,0,1,0) in the linked case.

We call a strategy *deterministic* (in either the linked or unlinked strategy space) if all its entries are either zero or one. It is *stochastic* if at least one entry is positive but smaller than one. We note that for an error rate $\varepsilon > 0$, any given strategy $\mathbf{p} = (p^k_{\mathbf{p}})$ translates into an *effective strategy* of the form $(1-\varepsilon)\mathbf{p} + \varepsilon(\mathbf{1}-\mathbf{p})$. For positive error rates it thus follows that everybody's effective strategy is stochastic.

Calculation of payoffs

When players use reactive strategies, their payoffs can be calculated explicitly. In the following, we present the respective algorithm for linked strategies. Payoffs for unlinked strategies can be calculated by using their representation (5) within the linked strategy space.

Suppose the two players use (effective) reactive strategies $\mathbf{p}, \tilde{\mathbf{p}} \in \mathcal{R}_L$, respectively. To calculate the players' payoffs, we represent the multichannel game as a Markov chain. The states of this Markov chain are the possible outcomes of a single round. If $\mathbf{a} = (a_1, \ldots, a_m) \in \{C, D\}^m$ is the respective action profile of the first player, and $\tilde{\mathbf{a}} = (\tilde{a}_1, \ldots, \tilde{a}_m) \in \{C, D\}^m$ is the action profile of the second player, the Markov chain's current state is given by $\omega := (\mathbf{a}, \tilde{\mathbf{a}})$. Given the Markov chain's current state, we can calculate the probability that its next state is $\omega' = (\mathbf{a}', \tilde{\mathbf{a}}')$ as

$$w_{\omega,\omega'} = \prod_{k=1}^{m} q^k_{\omega,\omega'} \cdot \tilde{q}^k_{\omega,\omega'}, \tag{10}$$

The individual factors in this product reflect each player's individual probability to make the required decision in each game,

$$q^k_{\omega,\omega'} = \begin{cases} p^k_{\tilde{\mathbf{a}}} & \text{if } a'_k = C \\ 1-p^k_{\tilde{\mathbf{a}}} & \text{if } a'_k = D, \end{cases} \qquad \text{and} \qquad \tilde{q}^k_{\omega,\omega'} = \begin{cases} \tilde{p}^k_{\mathbf{a}} & \text{if } \tilde{a}'_k = C \\ 1-\tilde{p}^k_{\mathbf{a}} & \text{if } \tilde{a}'_k = D. \end{cases} \tag{11}$$

By collecting all these products, we obtain the $2^{2m} \times 2^{2m}$ transition matrix $W = (w_{\omega,\omega'})$. For positive error rates, every entry of W is strictly positive. Therefore it follows from the Perron-Frobenius theorem that the time average of each outcome converges to the unique invariant distribution $\mathbf{v} = (v_\omega)$ of the transition matrix W. This invariant distribution can be found by solving the linear equation $\mathbf{v} = \mathbf{v}W$. Each entry v_ω of the invariant distribution gives the expected frequency to observe outcome $\omega = (\mathbf{a}, \tilde{\mathbf{a}})$ over all rounds of the multichannel game. Based on this invariant distribution, we can calculate marginal distributions for each of the m constituent games, by computing

$$v^k_{(a',\tilde{a}')} := \sum_\omega v_\omega \cdot e^k_\omega(a', \tilde{a}'). \tag{12}$$

Here, $e^k_\omega(a', \tilde{a}')$ is an indicator function. Its value is one if $\omega = (\mathbf{a}, \tilde{\mathbf{a}})$ with $\mathbf{a} = (a_1, \ldots, a_m)$ and $\tilde{\mathbf{a}} = (\tilde{a}_1, \ldots, \tilde{a}_m)$ satisfies $a_k = a'$ and $\tilde{a}_k = \tilde{a}'$. Otherwise its value is zero. The resulting marginal probabilities for each game k can be summarized in a vector $\mathbf{v}^k = (v^k_{CC}, v^k_{CD}, v^k_{DC}, v^k_{DD})$. The four entries represent the average abundance of observing each of the possible one-shot outcomes in game k. Using these marginal probabilities, we can calculate the players' expected payoff (3) as

$$\begin{aligned}\pi &= \sum_{k=1}^m \; v^k_{CC} \cdot R_k + v^k_{CD} \cdot S_k + v^k_{DC} \cdot T_k + v^k_{DD} \cdot P_k \\ \tilde{\pi} &= \sum_{k=1}^m \; v^k_{CC} \cdot R_k + v^k_{CD} \cdot T_k + v^k_{DC} \cdot S_k + v^k_{DD} \cdot P_k.\end{aligned} \tag{13}$$

Similarly, we can also compute the players' average cooperation rate in each game k as

$$\begin{aligned}\gamma_k &= v^k_{CC} + v^k_{CD} \\ \tilde{\gamma}_k &= v^k_{CC} + v^k_{DC}.\end{aligned} \tag{14}$$

For the simulation results shown in the main text and **SI**, we have implemented the above algorithm in MATLAB. The respective code is provided in the **Appendix**.

We note that one can derive a more explicit formula for the players' payoffs in multichannel games by using the technique of Press and Dyson[40]. To this end, suppose again the players use reactive strategies $\mathbf{p}$ and $\mathbf{q}$, and let $\mathbf{f} = (f_\omega)$ denote an arbitrary 2^{2m}-dimensional column vector indexed by ω. As in Press and Dyson, we define a real-valued function $D(\mathbf{p}, \mathbf{q}, \mathbf{f}) = \det(W')$. Here, $W' = (w'_{\omega,\omega'})$ is a matrix with entries

$$w'_{\omega,\omega'} = \begin{cases} w_{\omega,\omega'} & \text{if } \omega' \neq \omega \text{ and } \omega' \neq (D\ldots D;\; D\ldots D) \\ w_{\omega,\omega'} - 1 & \text{if } \omega' = \omega \text{ and } \omega' \neq (D\ldots D;\; D\ldots D) \\ f_\omega & \text{if } \omega' = (D\ldots D;\; D\ldots D), \end{cases} \tag{15}$$

with the values of $w_{\omega,\omega'}$ being defined as in Eq. (10). That is, for all but one column, the matrix W' corresponds to the matrix $W - I$ with I being the respective identity matrix. Only for the column that

corresponds to full mutual defection in the next round, the entries are replaced by the entries of $\mathbf{f}$. In the special case of a single game, $m=1$, this function thus becomes

$$D(\mathbf{p},\mathbf{q},\mathbf{f}) = \det \begin{pmatrix} p_C q_C - 1 & p_C(1-q_C) & (1-p_C)q_C & f_{CC} \\ p_D q_C & p_D(1-q_C) - 1 & (1-p_D)q_C & f_{CD} \\ p_C q_D & p_C(1-q_D) & (1-p_C)q_D - 1 & f_{DC} \\ p_D q_D & p_D(1-q_D) & (1-p_D)q_D & f_{DD} \end{pmatrix}. \tag{16}$$

We note that in Press and Dyson[40], this function is defined slightly differently, as

$$D(\mathbf{p},\mathbf{q},\mathbf{f}) = \det \begin{pmatrix} p_C q_C - 1 & p_C - 1 & q_C - 1 & f_{CC} \\ p_D q_C & p_D - 1 & q_C & f_{CD} \\ p_C q_D & p_C & q_D - 1 & f_{DC} \\ p_D q_D & p_D & q_D & f_{DD} \end{pmatrix}. \tag{17}$$

However, because the matrix in (17) can be obtained from the matrix in (16) by adding the first column to the second and third column (which does not change the determinant), the two functions coincide. By exactly the same argument as in Press and Dyson[40], it now follows that the players' payoffs π and $\tilde{\pi}$, as defined by Eq. (13), can be calculated with the formula

$$\pi = \frac{D(\mathbf{p},\mathbf{q},\mathbf{u})}{D(\mathbf{p},\mathbf{q},\mathbf{1})} \quad \text{and} \quad \tilde{\pi} = \frac{D(\mathbf{p},\mathbf{q},\tilde{\mathbf{u}})}{D(\mathbf{p},\mathbf{q},\mathbf{1})} \tag{18}$$

Here, $\mathbf{1}$ is the vector with all entries being equal to one; $\mathbf{u}=(u_\omega)$ is the vector that contains the one-round payoffs of player 1 for each state ω; and $\tilde{\mathbf{u}}=(\tilde{u}_\omega)$ is the corresponding vector for player 2. More formally, for a state $\omega=(\mathbf{a},\tilde{\mathbf{a}})$, the entries of u_ω and $\tilde{u}_\omega$ are defined by

$$u_\omega = \sum_{k=1}^{m} u^k_{a^k,\tilde{a}^k} \quad \text{and} \quad \tilde{u}_\omega = \sum_{k=1}^{m} \tilde{u}^k_{a^k,\tilde{a}^k}, \tag{19}$$

where $(u^k_{CC}, u^k_{CD}, u^k_{DC}, u^k_{DD}) = (R^k, S^k, T^k, P^k)$ and $(\tilde{u}^k_{CC}, \tilde{u}^k_{CD}, \tilde{u}^k_{DC}, \tilde{u}^k_{DD}) = (R^k, T^k, S^k, P^k)$. Two remarks are in order.

First, by adding up all columns in W' that correspond to a state ω' in which player 1 cooperates in game k in the next round, one obtains a column that only depends on $p^k_{\tilde{\mathbf{a}}}$. In particular, this column is then independent of the co-player's strategy. As in Press and Dyson[40], this result can be used to formally define zero-determinant strategies of multichannel games.

Second, as an algorithm to calculate payoffs, Eq. (18) has a similar computational complexity as Eq. (13). Because we also need the entries of the invariant distribution to compute additional quantities of interest (e.g., the average cooperation rate), we use Eq. (13) throughout our study.

Evolutionary dynamics

Description of the general dynamics. Herein, we take an evolutionary approach to multichannel games. Initially, players may pick arbitrary strategies that do not need to be optimal. However, over time they regularly revise their behaviors and adopt strategies that yield a better payoff. We aim to understand under which conditions the resulting strategy dynamics gives rise to cooperation.

To model the strategy dynamics, we study a pairwise comparison process[41,42] in a population of constant size N. We assume that initially all population members are unconditional defectors (using the respective representation of *ALLD* in the linked or the unlinked strategy space, depending on the considered condition). Then they engage in a multichannel game with every other population member to obtain a pairwise payoff as defined in Eq. (13). To get each player's overall payoff, we average over all the player's pairwise interactions with all other population members.

Players update their strategies in discrete time steps. In each time step, one of the population members is randomly drawn from the population. This player then gets the opportunity to update her strategy, either by random strategy exploration (corresponding to mutations in biological models) or by imitation (corresponding to selection). These two possible events are implemented as follows.

1. With probability μ (the mutation rate), the player engages in random strategy exploration. In that case, the player uniformly samples a strategy from the set of feasible strategies (either $\mathcal{R}_U$ or $\mathcal{R}_L$, depending on the considered condition). The player then adopts the randomly sampled strategy, and discards her previous strategy.

2. Alternatively, with probability $1-\mu$, the player considers imitating the strategy of someone else. To this end, the player randomly samples another player from the population, who serves as a potential role model. If the focal player's global payoff is π_F and the role model's payoff is π_R, the focal player adopts the role model's strategy with probability[43,44]

   $$\rho = \frac{1}{1+\exp\big[-s(\pi_R-\pi_F)\big]}. \tag{20}$$

   The parameter $s \geq 0$ reflects the strength of selection. It quantifies to which extent imitation is driven by relative payoff advantages. In one limiting case, $s \to 0$, payoffs are completely irrelevant. In this limit, the imitation probability simplifies to $1/2$ and imitation occurs purely at random. In the other limit $s \to \infty$, imitation events are strongly biased in favor of strategies that yield high payoffs. Here, the focal player only adopts the role model's strategy if it yields at least the focal player's previous payoff.

To quantify the evolutionary dynamics, we iterate the above described elementary updating events for many time steps. For positive mutation rates this yields an ergodic process on the space of all possible population compositions. We explore this process through simulations. For each time step t we compute the population's mean payoff $\hat{\pi}(t)$ and mean cooperation rate $\hat{\gamma}_k(t)$ in each game k, by averaging over all

players' interactions. If the simulation runs for T time steps in total, we define the simulation's average payoff and cooperation rate by

$$\hat{\pi} = \frac{1}{T}\sum_{t=1}^{T}\hat{\pi}(t) \quad \text{and} \quad \hat{\gamma}_k = \frac{1}{T}\sum_{t=1}^{T}\hat{\gamma}_k(t). \tag{21}$$

Evolution in the limit of rare mutations. The evolutionary process introduced above can be simulated more efficiently when mutations are rare[45–48]. As the mutation rate becomes sufficiently small, the population is typically homogeneous, such that every player uses the same strategy. Only occasionally a new mutant strategy is introduced into the population. This mutant strategy then either goes to extinction or fixes before the next mutant arises. The mutant's fixation probability can be computed explicitly[49]. For the imitation process considered herein, it is given by[50]

$$\rho_F = \frac{1}{1+\sum_{i=1}^{N-1}\prod_{j=1}^{i}\exp\Big[-s\big(\pi_M(j)-\pi_R(j)\big)\Big]}. \tag{22}$$

Here, $\pi_M(j)$ and $\pi_R(j)$ denote the mutant's and the resident's average payoff given that there are currently j mutants in the population.

We have simulated this rare-mutation process by consecutively introducing new mutant strategies into the population and determining their fate using the fixation probability (22). Each such simulation generates a sequence of resident strategies $\big(\mathbf{p}(0), \mathbf{p}(1), \dots\big)$. In this sequence, $\mathbf{p}(t)$ is the resident strategy after the t-th mutant has been introduced. Based on this sequence, we compute the corresponding mean payoffs over time $\big(\hat{\pi}(0), \hat{\pi}(1), \dots\big)$ and game-wise average cooperation rates $\big(\hat{\gamma}(0), \hat{\gamma}(1), \dots\big)$.

We use the above introduced rare mutation limit for all results shown in the main text, and for most of the results shown in the **SI**. However, as confirmed by **Supplementary Fig. 6**, similar results can be obtained for larger mutation rates as long as imitation events are sufficiently frequent to eliminate strategies with a low payoff from the population. The MATLAB code that we have used is provided in the **Appendix**.

Supplementary Note 3: Equilibrium analysis

Partners, semi-partners, and defectors

In the simulations shown in **Fig. 2**, individuals often adopt strategies that either cooperate in all games, cooperate in some games but defect in others, or strategies that do not cooperate at all. In the following, we aim to characterize the respective strategy classes of partners, semi-partners, and defectors formally.

To this end, we say a strategy is *self-cooperative* in game k if it induces two players with that strategy to mutually cooperate in the respective game. That is, if two players use the same self-cooperative strategy, we require the resulting cooperation rate to satisfy $\lim_{\varepsilon\to 0}\gamma_k = 1$. Similarly, we call a strategy

self-defective in game k if it gives rise to mutual defection: when applied by both players, it yields a limiting cooperation rate of $\lim_{\varepsilon\to 0}\gamma_k = 0$. A strategy is a *Nash equilibrium* if it is a best response to itself. Formally, if π is the payoff that each player gets when both players use the respective strategy, then any alternative payoff $\tilde{\pi}$ that could be obtained against that strategy satisfies $\tilde{\pi} \leq \pi$. These concepts allow us to formally introduce the different strategy classes highlighted in the main text.

Definition. *Consider an arbitrary strategy for a multichannel game with m games.*

1. *The strategy is a partner if it is a Nash equilibrium and if it is self-cooperative in each game.*
2. *The strategy is a game-k semi-partner if it is a Nash equilibrium and if it is self-cooperative in game k but self-defective in all other games.*
3. *The strategy is a defector if it is a Nash equilibrium and if it is self-defective in each game.*

It is important to stress that the above concepts are introduced generally. They do not require strategies to be reactive, nor do they make a distinction between linked or unlinked strategies. In particular, we do not only require a Nash equilibrium to be robust with respect to all deviations within the respective strategy class. Instead, we require it to be robust with respect to *all possible* deviations. In an equilibrium, players have no incentive to deviate even if they have access to more complex strategies that take into account arbitrarily long histories of past play. For the degenerate case that the multichannel game only consists of one game, the above definitions recover previous notions of partners[51–53] (also called 'good strategies') and defectors[54–57] in repeated two-player games.

Characterization of Nash equilibria in multichannel donation games

In many natural instances, cooperation means to pay a cost $c > 0$ in order to generate a benefit $b > c$ for the co-player. The four possible payoffs are then given by $R = b - c$, $S = -c$, $T = b$, and $P = 0$. Social dilemmas of this form are called donation games[31]. Because the resulting payoffs satisfy the payoff inequalities $T > R > P > S$, any donation game is a prisoner's dilemma.

In the following, we characterize partners, semi-partners, and defectors for the case that players interact across m donation games. To simplify notation in what follows, it is useful to introduce an indicator function $e^k_{\mathbf{a}}$. For a given action profile $\mathbf{a} = (a_1, \ldots, a_m)$, the value of this indicator function is one if $a_k = C$ and it is zero if $a_k = D$.

Proposition 1 (Partner strategies in the linked case)**.**
Consider a multichannel game consisting of m donation games, where $b_k > c_k$ is the benefit and cost of cooperation in game k, respectively. For a strategy $\mathbf{p} \in \mathcal{R}_L$ that is self-cooperative in each game the following two are equivalent:

1. *The strategy $\mathbf{p}$ is a partner.*

2. *The following inequalities are satisfied for all* 2^m *possible action profiles* $\mathbf{a}\in\{C,D\}^m$,

$$\sum_{k=1}^{m} b_k \cdot (1-p_{\mathbf{a}}^k) \geq \sum_{k=1}^{m} c_k\cdot(1-e_{\mathbf{a}}^k). \tag{23}$$

All proofs are provided in the **Appendix**. The 2^m inequalities in (23) provide upper bounds on how cooperative players are allowed to be off the equilibrium path.

Proposition 1 implies that the set of partner strategies is always non-empty. To see this, we note for $\mathbf{a} = (C, \ldots, C)$ that the right hand's side of (23) becomes zero and hence imposes no restriction. Otherwise, for $\mathbf{a} \neq (C, \ldots, C)$ we can simply choose $p_{\mathbf{a}}^k$ sufficiently small, such that the left hand's side is approximately equal to $\sum b_k$, which by definition exceeds $\sum c_k$.

To gain some further insights on partner strategies, we analyze how the left and the right hand's side of condition (23) depend on the game parameters. For a given co-player's action profile $\mathbf{a}$, we say a parameter change allows a player to be more lenient in game k if it allows the player to increase her value of $p_{\mathbf{a}}^k$ while still satisfying the above inequalities. We find that the following factors allow a strategy to be more lenient in game k.

(i) A large benefit and small costs of cooperation: Larger values of b_k tend to increase the left hand's side of condition (23), whereas smaller costs tend to decrease the right hand's side.

(ii) A cooperative co-player: The more cooperative the co-player has been in the previous round, the fewer summands are there on the right hand's side of condition (23).

(iii) The focal player is less lenient in other games: The lower $p_{\mathbf{a}}^j$ for $j \neq k$, the more easily condition (23) is satisfied even for large $p_{\mathbf{a}}^k$.

The third aspect is particularly noteworthy. It suggests that the focal player may choose to some extent in which game she wants to punish a defecting co-player. In particular, she is not required to respond in the very same game. Instead her strategy is most likely to meet the conditions for being a partner if it prescribes to retaliate in some high-stake game instead: To meet the inequalities in (23), a player's conditional cooperation probability $p_{\mathbf{a}}^k$ should be particularly small in those games with a large benefit b_k.

Similarly to partners, we can characterize defectors and semi-partners.

Proposition 2 (Defectors in the linked case)**.**
Consider a multichannel game that consists of m *donation games, and let* $\mathbf{p}\in\mathcal{R}_L$ *be self-defective in all games. Then the following are equivalent.*

1. *The strategy* $\mathbf{p}$ *is a defector.*
2. *The following inequalities are satisfied for all* 2^m *possible action profiles* $\mathbf{a}\in\{C,D\}^m$,

$$\sum_{k=1}^{m} b_k \cdot p_{\mathbf{a}}^k \leq \sum_{k=1}^{m} c_k \cdot e_{\mathbf{a}}^k. \tag{24}$$

Proposition 3 (Semi-partners in the linked case)**.**
Consider a multichannel game that consists of m donation games. Let the strategy $\mathbf{p} \in \mathcal{R}_L$ be self-cooperative in game k but self-defective in all other games. Then the following are equivalent.

1. *The strategy $\mathbf{p}$ is a game k semi-partner.*
2. *The following inequalities are satisfied for all 2^m possible action profiles $\mathbf{a} \in \{C, D\}^m$,*

$$b_k \cdot (1-p_{\mathbf{a}}^k) - c_k \cdot (1-e_{\mathbf{a}}^k) \geq \sum_{l \neq k} b_l p_{\mathbf{a}}^l - \sum_{l \neq k} c_l e_{\mathbf{a}}^l. \tag{25}$$

It is instructive to discuss these strategy classes in simple special cases.

Example (Partners, semi-partners, and defectors when there is either one or two games)**.**

1. For $m=1$, Proposition 1 implies that a reactive strategy is a partner if and only if

$$p_C^1 = 1 \quad \text{and} \quad 0 < p_D^1 \leq 1 - c_1/b_1. \tag{26}$$

   The first equation and the first inequality are required for the strategy to be self-cooperative. The second inequality is required for the strategy to be a Nash equilibrium. In particular, we note that the strategy Tit-for-Tat (p_C^1, p_D^1)=(1,0) is excluded from the set of partners. Tit-for-Tat is not self-cooperative because errors can easily destabilize mutual cooperation even as the error rate becomes arbitrarily small[39,58].

   The partner strategies described above coincide with the stable cooperative strategies described in earlier work on the repeated prisoner's dilemma[31]. In particular, the limiting strategy where p_D^1 takes its maximum value is known as the strategy Generous Tit-for-Tat[39,58].

   Similarly, we can use Proposition 2 to characterize the reactive strategies that are defectors,

$$0 \leq p_C^1 \leq c/b \quad \text{and} \quad p_D^1 = 0. \tag{27}$$

   Again, these conditions agree with previous descriptions of stable self-defecting strategies in the repeated prisoner's dilemma[55].

2. For $m=2$, a partner strategy needs to satisfy

$$\begin{aligned} p_{CC}^1 = p_{CC}^2 &= 1 \\ b_1(1-p_{CD}^1) + b_2(1-p_{CD}^2) &\geq c_2 \\ b_1(1-p_{DC}^1) + b_2(1-p_{DC}^2) &\geq c_1 \\ b_1(1-p_{DD}^1) + b_2(1-p_{DD}^2) &\geq c_1 + c_2. \end{aligned} \tag{28}$$

   The first two equalities follow from the requirement that the strategy is self-cooperative. The other three inequalities guarantee that a co-player's defection in either of the two games is punished with sufficiently high likelihood. The conditions in Eq. (28) are reproduced in Eq. (5) of the main text.

Similarly, according to Proposition 2 defectors need to meet the following conditions

$$\begin{aligned} b_1\, p_{CC}^1 + b_2\, p_{CC}^2 &\le c_1 + c_2 \\ b_1\, p_{CD}^1 + b_2\, p_{CD}^2 &\le c_1 \\ b_1\, p_{DC}^1 + b_2\, p_{DC}^2 &\le c_2 \\ p_{DD}^1 = p_{DD}^2 &= 0. \end{aligned} \tag{29}$$

In this case, the first three inequalities ensure that a defector is not too cooperative in response to a co-player's cooperation in the previous round. The last two equalities follow from the requirement that mutual defection needs to be an absorbing state.

Finally, a game-1 semi-partner is required to meet the following conditions,

$$\begin{aligned} b_1(1-p_{CC}^1) &\ge b_2\, p_{CC}^2 - c_2 \\ p_{CD}^1 = 1,\, p_{CD}^2 &= 0 \\ b_1(1-p_{DC}^1) - c_1 &\ge b_2\, p_{DC}^2 - c_2 \\ b_1(1-p_{DD}^1) - c_1 &\ge b_2\, p_{DD}^2 \end{aligned} \tag{30}$$

Again, the two equalities make cooperation in game 1 and defection in game 2 an absorbing state, whereas the inequalities ensure that players do not have an incentive to deviate. The corresponding characterization of game-2 semi-partners takes an analogous form.

For the special case of a multichannel game with $b_1 = 4$, $b_2 = 3/2$ and $c_1 = c_2 = 1$, the respective conditions (28) – (30) for partners, semi-partners and defectors are illustrated in **Supplementary Fig. 2**.

Because the linked strategy space contains the unlinked strategies as a special case, the above characterizations also allow us to identify the partners, semi-partners, and the defectors in the unlinked case.

Proposition 4 (Partners, semi-partners, and defectors in the unlinked case)**.**
Consider a multichannel game that consists of m donation games and suppose $\mathbf{p} \in \mathcal{R}_U$.

1. *The strategy $\mathbf{p}$ is a partner if and only if it is self-cooperative and $p_D^k \le 1 - c_k/b_k$ for all games k.*
2. *It is a defector if and only if it is self-defective and if $p_C^k \le c_k/b_k$ for all k.*
3. *It is a game-k semi-partner if and only if it is self-cooperative in game k, self-defective in all other games, and if $p_D^k \le 1 - c_k/b_k$ and $p_C^l \le c_l/b_l$ for all $l \ne k$.*

For the special case of a multichannel game consisting of two donation games with $b_1 = 4$, $b_2 = 3/2$ and $c_1 = c_2 = 1$, the conditions of Proposition 4 are illustrated in **Supplementary Fig. 1**. Notably, the respective inequalities make restrictions for each individual cooperation probability p_a^k. This is in contrast to the linked case, where players are allowed to be more lenient in one game if only they are sufficiently responsive in another game, as expressed by the sums in Eqs. (23) – (25). This again reflects the key

idea that only when games are linked, players can use cooperation in one game to enforce cooperation in another. This becomes even more transparent when the same payoff matrix is used for every game.

Example (Multichannel games with m identical games)**.**
Suppose the two players interact across m identical donation games, and let b and c denote the respective benefit and cost of cooperation in each game. To be a partner in the unlinked strategy space, it follows from Proposition 4 that the strategy needs to satisfy

$$p_C^k = 1 \quad \text{and} \quad p_D^k \leq 1 - c/b \quad \text{for every game } k. \tag{31}$$

In contrast, the respective conditions in the linked strategy space become

$$\begin{aligned} p_{\mathbf{a}}^k &= 1 && \text{for all games } k \text{ and } \mathbf{a} = (C, \ldots, C), \\ \frac{1}{m}\sum_{k=1}^{m} p_{\mathbf{a}}^k &\leq 1 - \frac{m_{\mathbf{a}}^D}{m}\frac{c}{b} && \text{for all action profiles } \mathbf{a} \neq (C, \ldots, C), \end{aligned} \tag{32}$$

where $m_{\mathbf{a}}^D$ denotes the number of D's in action profile $\mathbf{a}$. According to conditions (32), a player does not need to ensure that each of her conditional cooperation probabilities is below some threshold. However, she needs to ensure that the threshold is met *on average*, when summing up over all m games.

Relative abundance of partners, semi-partners and defectors

The conditions derived in Propositions 1 and 4 also allow us to calculate how abundant partners are among all fully self-cooperative strategies. To this end, consider a randomly chosen unlinked strategy $\mathbf{p} \in \mathcal{R}_U$ that is self-cooperative in all games, such that $p_C^k = 1$ for all k. For this strategy to be a partner (to be a Nash equilibrium), Proposition 4 requires that all cooperation probabilities after mutual defection need to satisfy $p_D^k \leq 1 - c_k/b_k$. Hence, the probability that a randomly chosen fully self-cooperative strategy is a partner is given by

$$f_U^P = \prod_{k=1}^{m} \left(1 - \frac{c_k}{b_k}\right). \tag{33}$$

Similarly we can compute the relative abundance of partners within the space of linked strategies. To this end, take a randomly chosen strategy $\mathbf{p} \in \mathcal{R}_L$ that is self cooperative in each game, such that $p_{(C,\ldots,C)}^k = 1$ for all k. According to Proposition 1, the probability that this strategy is a partner is

$$f_L^P = \prod_{\mathbf{a} \in \{C,D\}^m} \left(\int_{[0,1]^m} H\Big(\sum_{k=1}^{m} b_k \cdot (1 - p_{\mathbf{a}}^k) - \sum_{k=1}^{m} c_k \cdot (1 - e_{\mathbf{a}}^k) \Big) dp_{\mathbf{a}}^1 \ldots dp_{\mathbf{a}}^m \right). \tag{34}$$

Here, $H(x)$ is the Heaviside function, such that $H(x) = 1$ if $x \geq 0$ and $H(x) = 0$ if $x < 0$. Again, it is instructive to explore these relative abundances for low-dimensional examples.

Example (Relative abundance of partners for $m = 1$ and $m = 2$)**.**

1. If there is only one game, the linked and the unlinked case result in the same relative abundance of partners, $f_U^P = f_L^P = 1 - c_1/b_1$, as one may expect.

2. If there are two games, the relative abundances of partners differ between the linked and the unlinked case. For simplicity, we assume in the following that both games have the same cooperation costs, $c_1 = c_2 =: c$. In the unlinked case, it then follows from Eq. (33) that the relative abundance of partners is

$$f_U^P = \left(1 - \frac{c}{b_1}\right)\left(1 - \frac{c}{b_2}\right). \tag{35}$$

In the linked case, the relative abundance of partners is a product of four factors (corresponding to the four possible action profiles $\mathbf{a}$ used by the co-player across the two games),

$$f_L^P = g_{CC} \cdot g_{CD} \cdot g_{DC} \cdot g_{DD}. \tag{36}$$

For each of the four factors, we need to evaluate the respective integral in Eq. (34), which yields

$$g_{CC} = 1, \quad g_{CD} = g_{DC} = 1 - \frac{1}{2} \cdot \frac{c}{b_1} \cdot \frac{c}{b_2}, \quad g_{DD} = \begin{cases} 1 - 2\frac{c}{b_1} \cdot \frac{c}{b_2} & \text{if } b_1, b_2 > 2c \\ \frac{(b_1 + b_2 - 2c)^2}{2 b_1 b_2} & \text{if } b_1, b_2 < 2c \\ 1 - \frac{4c - \min(b_1, b_2)}{2\max(b_1, b_2)} & \text{otherwise.} \end{cases} \tag{37}$$

Geometrically, the latter three factors correspond to the colored areas in the bottom three panels of **Supplementary Fig. 2a**.

In **Supplementary Fig. 3a**, we illustrate the relative abundances f_U^P and f_L^P, as given by Eqs. (35) and (36). The top panel indicates that for $b_2 = 3/2$ and $c = 1$, partners are relatively more abundant in the linked case, for any value of b_1. Moreover, the difference in the abundance of partners between linked and unlinked increases with the benefit in the first game. In **Supplementary Fig. 3b**, we show a similar result when b_1 and b_2 are varied simultaneously. Especially if there is a high benefit in one game but a comparably low benefit in the other, partner strategies are much more abundant in the linked case.

The relative abundance of semi-partners and defectors can be computed analogously to the case of partners, by integrating over all reactive strategies that satisfy the respective conditions in Propositions 2 – 4. In **Supplementary Fig. 3**, we again depict these relative abundances for the special case of a multichannel game consisting of two donation games. In particular, the figure shows that in the linked case, defectors are relatively rare. Moreover, their abundance decreases even further in multichannel games in which at least one game yields a high benefit.

Numerical identification of strategies

Within the entire set of reactive strategies, the subsets of partners, semi-partners, and defectors is vanishingly small. For example, to be self-cooperative, partner strategies require the cooperation probability

after mutual cooperation to be exactly one. However, if the respective cooperation probability is generated at random, by uniformly sampling a number from the unit interval, the likelihood of observing such a mutant is zero.

In order to quantify the abundance of the different strategy classes in our evolutionary simulations, we have thus considered approximate notions of partners, semi-partners and defectors. For the simulations, we say that a strategy is approximately self-cooperative if its cooperation rate against itself is at least $1-\theta$, for some threshold $\theta > 0$. Similarly, the strategy is approximately self-defective if its cooperation rate is at most θ. A strategy is then classified as a partner, semi-partner and a defector, if it meets the respective (exact) inequalities in Propositions 1 – 4, and if it is approximately self-cooperative or self-defective, respectively. For the figures **Fig. 3**, **Supplementary Fig. 4**, and **Supplementary Fig. 5**, we have used a threshold $\theta = 0.2$. As shown in **Fig. 3**, this threshold ensures that the sets of approximate partners, semi-partners, and defectors are small compared to the space of all reactive strategies.

Supplementary Note 4: Model extensions

Memory-1 strategies

In the previous sections, we have explored the evolution of cooperation assuming that players can choose among all reactive strategies. This strategy class assumes that when players make their decisions, they only respond to the co-player's actions in the previous round. Previous research on the repeated prisoner's dilemma suggests that cooperation can be further stabilized if players are additionally allowed to take their own previous actions into account[59–64]. In the following, we thus introduce such *memory-1 strategies* for multichannel games. For the linked case we define a memory-1 strategy to be an element of the set

$$\mathcal{M}_L = \Big\{ \mathbf{p} = (p^k_\omega)_{\omega\in\{C,D\}^m\times\{C,D\}^m,\ k\in\{1,\ldots,m\}} \ \Big|\ p^k_\omega \in [0,1] \ \text{ for all } \omega \text{ and all } k \Big\}. \qquad (38)$$

Such strategies describe for each game k with which probability p^k_ω to cooperate; this probability depends on the outcome $\omega = (\mathbf{a}, \tilde{\mathbf{a}})$ of the previous round, where $\mathbf{a}$ is the focal player's previous action profile, and $\tilde{\mathbf{a}}$ is the co-player's previous action profile. It follows that the elements of $\mathcal{M}_L$ are $m \cdot 2^{2m}$-dimensional vectors with entries in the unit interval. Similarly, we can also define memory-1 strategies for the unlinked case as elements of the set

$$\mathcal{M}_U = \Big\{ \mathbf{p} = (p^k_{(a_k,\tilde{a}_k)})_{a,\tilde{a}_k\in\{C,D\},\ k\in\{1,\ldots,m\}} \ \Big|\ p^k_{(a_k,\tilde{a}_k)} \in [0,1] \ \text{ for all } a_k, \tilde{a}_k \text{ and all } k \Big\}. \qquad (39)$$

Here, the player's cooperation probability $p^k_{(a_k,\tilde{a}_k)}$ in game k only depends on the players' previous actions in the respective game. It follows that $\mathcal{M}_U$ corresponds to the $4m$ dimensional unit cube.

Again, it is instructive to consider examples.

1. For $m = 1$, the spaces of unlinked and linked memory-1 strategies coincide. Each memory-1 strategy takes the form $\mathbf{p} = (p^1_{CC}, p^1_{CD}, p^1_{DC}, p^1_{DD})$, which recovers the usual definition of these strategies in the repeated prisoner's dilemma[31]. As an example, the classical strategy Win Stay Lose Shift[59,60] takes the form (1,0,0,1). That is, it cooperates if and only if either both players cooperated or they both defected.

2. For $m=2$, unlinked memory-1 strategies are 8-dimensional vectors that take the form $\mathbf{p} = (p^1_{CC}, p^1_{CD}, p^1_{DC}, p^1_{DD};\ p^2_{CC}, p^2_{CD}, p^2_{DC}, p^2_{DD})$. Here, $p^k_{a\tilde{a}}$ denotes the player's cooperation probability in game k, given that the player herself used action a in the previous round whereas the co-player used action $\tilde{a}$ in that game. A memory-1 strategy in the linked strategy space is now a 32-dimensional vector with entries $p^k_{(a_1a_2;\ \tilde{a}_1\tilde{a}_2)}$. Here, a_1, a_2 denote the focal player's previous actions in games 1 and 2, whereas $\tilde{a}_1, \tilde{a}_2$ denote the co-player's previous actions.

Payoffs for a game between two players with (linked) memory-1 strategies $\mathbf{p}$ and $\tilde{\mathbf{p}}$ can be computed analogously to the case of reactive strategies, as detailed in the respective Section "Calculation of payoffs". Again, we can represent the game as a Markov chain with transition probabilities $w_{\omega,\omega'} = \prod_k q^k_{\omega,\omega'}\tilde{q}^k_{\omega,\omega'}$. As the only difference, the individual factors now need to be defined as

$$q^k_{\omega,\omega'} = \begin{cases} p^k_\omega & \text{if } a'_k = C \\ 1-p^k_\omega & \text{if } a'_k = D, \end{cases} \quad \text{and} \quad \tilde{q}^k_{\omega,\omega'} = \begin{cases} \tilde{p}^k_{\tilde{\omega}} & \text{if } \tilde{a}'_k = C \\ 1-\tilde{p}^k_{\tilde{\omega}} & \text{if } \tilde{a}'_k = D. \end{cases} \tag{40}$$

Here, $\omega = (\mathbf{a}, \tilde{\mathbf{a}})$ is the previous round's outcome from the perspective of player 1, and $\tilde{\omega} = (\tilde{\mathbf{a}}, \mathbf{a})$ is the previous round's outcome from the perspective of player 2. By calculating the invariant distribution of the transition matrix $W = (w_{\omega,\omega'})$, we can again calculate the player's payoffs and their average cooperation rates in each game.

Based on the previous literature, we expected that evolutionary simulations would lead to strategies similar to the win-stay lose-shift behaviors observed in repeated games[59–63]. In the following, let us thus define two different notions of such strategies for multichannel games.

Definition (*WSLS* and *CIC*)**.** *Consider a multichannel game with m games.*

1. *We define WSLS as the strategy that cooperates in game k if and only if both players previously used the same action in that game. That is, for the respective version $\mathbf{p} \in \mathcal{M}_U$ in the unlinked strategy space we require*

$$p^k_{(a_k,\tilde{a}_k)} = \begin{cases} 1 & \text{if } a_k = \tilde{a}_k \\ 0 & \text{if } a_k \neq \tilde{a}_k. \end{cases} \tag{41}$$

2. *We define CIC to be the strategy that cooperates in game k if and only if both players have previ-*

ously used the same action profile. This strategy only exists in $\mathcal{M}_L$, and it requires

$$p^k_{(\mathbf{a},\tilde{\mathbf{a}})} = \begin{cases} 1 & \text{if } \mathbf{a}=\tilde{\mathbf{a}} \\ 0 & \text{if } \mathbf{a}\neq\tilde{\mathbf{a}}. \end{cases} \tag{42}$$

The strategy *CIC* is motivated by previous characterizations of *All-or-none* strategies[63,64]. It only prescribes to cooperate if players use the same action in each considered game. We note that while *WSLS* allows players to cooperate in some games but not in others, *CIC* players perfectly synchronize their behavior across the m games. Unless there is an error, they cooperate in one game in a given round if and only if they cooperate in all of them.

Both strategies, *WSLS* and *CIC*, are self-cooperative. Due to their definition, they continue to cooperate in all games if all players have already cooperated in the previous round. But even if one of the players defects by error, the two strategies quickly revert to mutual cooperation. For example, if both players adopt *WSLS* and one player defects in game k, then both players defect in that game in the next round, after which they recover mutual cooperation.

To explore when *WSLS* and *CIC* are partners, we thus only need to characterize under which condition they are Nash equilibria. In the following, we use a slightly stronger notion of equilibrium, subgame perfection[65]. Whereas Nash equilibrium requires that a strategy does not allow for a profitable deviation given that every co-player acts as required, a subgame perfect equlibrium must not exhibit any profitable deviation after *any* history (even those histories off the equilibrium path). In particular, every subgame perfect equilibrium is a Nash equilibrium, but the converse does not need to hold[66].

Proposition 5 (Stability of *WSLS* and *CIC*)**.**
Consider a multichannel game consisting of m donation games, and let the benefit and cost of cooperation in each game k be b_k and c_k.

1. *WSLS is a subgame perfect equilibrium if and only if $b_k \geq 2c_k$ for all k.*
2. *CIC is a subgame perfect equilibrium if and only if $\sum_k b_k \geq 2\sum_k c_k$.*

Several remarks are in order.

1. Formally, Proposition 5 only guarantees that no other strategy has a selective advantage to invade either *CIC* or *WSLS* when the respective conditions are met. However, this does not rule out neutral invasions that undermine the two equilibria[33,34,67]. However, if the inequalities in Proposition 5 are strict, we actually show a stronger result. In that case, the two strategies are in fact evolutionarily stable provided that the error rate is positive but sufficiently small.
2. When the above conditions are met, *CIC* and *WSLS* are stable against all possible mutant strategies. Our proof does not require that mutants themselves are restricted to memory-1 strategies.

3. According to Proposition 5, *CIC* makes it easier to sustain cooperation. While *WSLS* requires that the benefit of cooperation is at least twice the cost in every single game, *CIC* only requires this to be true on average, across all games. In particular, if *WSLS* is an equilibrium for a given multichannel game, then so is *CIC*. The two strategies are equivalent in terms of their ability to sustain cooperation only if all games are identical. In that case, both strategies require $b > 2c$, where b and c are now the benefit and cost of cooperation in every single game. This observation suggests that linking the two games should be particularly valuable in multichannel games in which the games differ from each other.

In **Fig. 4**, we show simulations for the special case that the multichannel game consists of two donation games with $b_1 = 4$, $b_2 = 2$, and $c_1 = c_2 = 1$ for a positive error rate $\varepsilon = 0.01$. The numerical results reflect the above analytical findings in the following sense:

(i) For the above parameters, *CIC* is evolutionarily stable, whereas *WSLS* is not. In line with this, we observe more cooperation when players are allowed to link the two games.

(ii) In the unlinked case, strategies are consistent with *WSLS* in the first game: players are most likely to cooperate if either both players cooperated in the previous round in that game, or if they both defected. In the second game, behavior rather resembles a GRIM strategy: players cooperate after mutual cooperation, but they defect otherwise.

(iii) In the linked case, average behavior reflects *CIC* in the sense that players are most cooperative if both players acted in the same way in the previous round. Moreover, behavior in the two games seems to be largely synchronized: the conditional cooperation probabilities in the first game are similar to the conditional cooperation probabilities in the second game. This observation further supports our finding that players tend to link the different games when given the chance.

Different types of games

To illustrate our framework, we have assumed in previous sections that the different games take the form of a donation game. In evolutionary game theory, the donation game serves as an important baseline model. It has the useful mathematical property of *equal gains from switching*[68], meaning that payoffs satisfy $R + P = T + S$. This payoff relationship implies that the value of cooperation to the co-player is independent of whether the co-player herself cooperated. This property is useful for two reasons.

(i) Equal gains from switching simplify the proofs of Propositions 1 – 4. When this property holds, a player's payoff in game k only depends on her own average cooperation rate γ_k and on her co-player's rate $\tilde{\gamma}_k$. Without equal gains from switching, we need to take correlations between the players' cooperation rates into account, by computing the marginal distributions v^k_{CC}, v^k_{CD}, v^k_{DC}, and v^k_{DD}.

(ii) Because $R > P$, equal gains from switching imply $2R > T{+}S$. This means that mutual cooperation in every round is the collectively optimal outcome for the group. If instead $2R < T + S$, players can achieve higher payoffs by taking turns; for example, player 1 cooperates in all odd rounds, whereas player 2 cooperates in all even rounds. Such games require a different notion of partners: A partner strategy now should maintain alternating cooperation between the two players, instead of enforcing mutual cooperation in every round.

Providing a formal analysis for arbitrary social dilemmas is beyond the scope of this work, but we can easily apply our framework to simulate such multichannel games. In **Supplementary Fig. 9** and **Supplementary Fig. 10**, we present several examples, based on the snowdrift game, the volunteer's dilemma, and the sculling game.

The snowdrift game[69,70] captures situations in which cooperators benefit from their own actions, and where players share the costs if they both cooperate. Payoffs are thus given by the payoff matrix

$$\begin{array}{c} \\ C \\ D \end{array} \begin{array}{c} \begin{array}{cc} C & D \end{array} \\ \left(\begin{array}{cc} b - \frac{c}{2} & b - c \\ b & 0 \end{array} \right) \end{array}. \tag{43}$$

Again, $c > 0$ and $b > c$ are the cost and the benefit of cooperation, respectively. As in the donation game, players have an incentive to defect against an unconditional cooperator. However, against an unconditional defector, players prefer to cooperate. We note that the snowdrift game satisfies the equality $2R{=}T{+}S$. Players who aim to optimize social welfare are thus indifferent between mutually cooperating in every round and cooperating in turns.

The volunteer's dilemma[71] has a similar structure as the snowdrift game, but now two cooperators have to pay the full cost,

$$\begin{array}{c} \\ C \\ D \end{array} \begin{array}{c} \begin{array}{cc} C & D \end{array} \\ \left(\begin{array}{cc} b - c & b - c \\ b & 0 \end{array} \right) \end{array}. \tag{44}$$

In particular, this game satisfies $2R < T + S$. Players now strictly prefer to take turns when cooperating, rather than mutually cooperating in every round.

Finally, we consider a variant of the sculling game[72]. Here, the benefit that each player receives depends non-monotonically on the number of cooperators. We use the payoff matrix

$$\begin{array}{c} \\ C \\ D \end{array} \begin{array}{c} \begin{array}{cc} C & D \end{array} \\ \left(\begin{array}{cc} (1{+}\alpha)b{-}c & \alpha b{-}c \\ \alpha b & 0 \end{array} \right) \end{array}. \tag{45}$$

Assuming that the factor α satisfies $0 < \alpha < c/b$ and that $b > c > 0$, we obtain a coordination game. Each player prefers to choose the same action as the other player. In particular, mutual cooperation can now be sustained even in a single instance of the game. Moreover, mutual cooperation is risk-dominant if

$c/b < (1+\alpha)/2$.

In **Supplementary Fig. 9** we consider scenarios in which players simultaneously engage in two different versions of each game type. In **Supplementary Fig. 10**, we additionally study the dynamics when each game type is coupled with a donation game. For the first two game types, the snowdrift game and the volunteer's dilemma, we recover our previous result that players achieve higher payoffs by linking their games. Interestingly, however, when players engage in a volunteer's dilemma, their payoffs do not approach the mutual optimum even as the benefit parameter b becomes large. These results suggest that to coordinate successfully, and to switch between cooperation and defection in an alternating fashion, players need to have more than one-round memory. For the sculling game, we observe that linking can sometimes reduce the players' payoffs. Such a disadvantage of linkage is most pronounced when the benefit parameter becomes large (such that cooperation becomes risk-dominant already in the one-shot game). In those cases, players achieve almost full cooperation in the sculling game already in the unlinked case. These findings suggest that linkage is most beneficial in strict social dilemmas, where repeated interactions are necessary for cooperation to evolve.

Supplementary Note 5: Appendix

Proofs of the equilibrium results

Partners, semi-partners, and defectors. In the following we derive the characterizations of partners, semi-partners, and defectors within the space of reactive strategies. To this end, let us recall some notation. For a game between two (arbitrary) players engaged in a multichannel game, we denote by $\mathbf{v} = (v_\omega)$ the resulting outcome distribution. It describes, for each outcome $\omega = (\mathbf{a}, \tilde{\mathbf{a}})$ how often that outcome is observed on average over the course of the infinitely repeated game (assuming that these averages converge). For a given invariant distribution $\mathbf{v}$, we define marginal distributions that describe how often we are to observe each player's individual action profile,

$$v_{\mathbf{a}} := \sum_{\tilde{\mathbf{a}} \in \{C,D\}^m} v_{(\mathbf{a},\tilde{\mathbf{a}})} \qquad \text{and} \qquad \tilde{v}_{\tilde{\mathbf{a}}} := \sum_{\mathbf{a} \in \{C,D\}^m} v_{(\mathbf{a},\tilde{\mathbf{a}})} \tag{46}$$

Finally, we use γ_k and $\tilde{\gamma}_k$ to denote the average frequency with which each player cooperates in game k. This frequency is defined by Eq. (14), or equivalently by

$$\gamma_k = \sum_{\mathbf{a} \in \{C,D\}^m} v_{\mathbf{a}} e^k_{\mathbf{a}} \qquad \text{and} \qquad \tilde{\gamma}_k = \sum_{\tilde{\mathbf{a}} \in \{C,D\}^m} \tilde{v}_{\tilde{\mathbf{a}}} e^k_{\tilde{\mathbf{a}}}. \tag{47}$$

Analogously, the players' defection probabilities in game k are given by

$$1-\gamma_k = \sum_{\mathbf{a} \in \{C,D\}^m} v_{\mathbf{a}} (1-e^k_{\mathbf{a}}) \qquad \text{and} \qquad 1-\tilde{\gamma}_k = \sum_{\tilde{\mathbf{a}} \in \{C,D\}^m} \tilde{v}_{\tilde{\mathbf{a}}} (1-e^k_{\tilde{\mathbf{a}}}). \tag{48}$$

To characterize partners, semi-partners, and rivals, we first introduce a relationship between a player's

reactive strategy and the resulting invariant distribution in a game against an arbitrary co-player. Because a relationship of this kind has first been described by Akin in the context of a repeated prisoner's dilemma[52], we call it Akin's Lemma.

Lemma (Akin's Lemma)**.**
Consider a multichannel games with m games. Suppose player 1 applies a reactive strategy $\mathbf{p}\in\mathcal{R}_L$ whereas player 2 applies an arbitrary strategy, and suppose the resulting average distribution to observe each outcome is given by $\mathbf{v}$. Then for each game k, player 1's cooperation rate satisfies

$$\gamma_k = \sum_{\tilde{\mathbf{a}}\in\{C,D\}^m} \tilde{v}_{\tilde{\mathbf{a}}}\cdot p^k_{\tilde{\mathbf{a}}}. \tag{49}$$

Equivalently, the player's defection rate satisfies

$$1-\gamma_k = \sum_{\tilde{\mathbf{a}}\in\{C,D\}^m} \tilde{v}_{\tilde{\mathbf{a}}}\cdot(1-p^k_{\tilde{\mathbf{a}}}). \tag{50}$$

Proof. Given the strategies of the two players, let $\mathbf{v}(t)=\big(v_\omega(t)\big)$ denote the expected outcome distribution in round t. We can then define player 1's expected probability to cooperate in game k in round t as

$$\gamma_k(t) := \sum_{\omega=(\mathbf{a},\tilde{\mathbf{a}})} v_\omega(t)\cdot e^k_{\mathbf{a}}. \tag{51}$$

Because player 1 applies a reactive strategy, we can compute her probability to cooperate in the subsequent round as

$$\gamma_k(t+1) = \sum_{\omega=(\mathbf{a},\tilde{\mathbf{a}})} v_\omega(t)\cdot p^k_{\tilde{\mathbf{a}}}. \tag{52}$$

Hence the difference in cooperation probabilities can be written as

$$\Delta_k(t) := \gamma_k(t+1)-\gamma_k(t) = \sum_{\omega=(\mathbf{a},\tilde{\mathbf{a}})} v_\omega(t)\cdot(p^k_{\tilde{\mathbf{a}}}-e^k_{\mathbf{a}}). \tag{53}$$

By summing up over the first τ rounds and averaging, we obtain

$$\frac{1}{\tau}\sum_{t=1}^{\tau}\Delta_k(t) = \frac{1}{\tau}\sum_{t=1}^{\tau}\sum_{\omega} v_\omega(t)\cdot(p^k_{\tilde{\mathbf{a}}}-e^k_{\mathbf{a}}) = \sum_{\omega}(p^k_{\tilde{\mathbf{a}}}-e^k_{\mathbf{a}})\left(\frac{1}{\tau}\sum_{t=1}^{\tau}v_\omega(t)\right). \tag{54}$$

On the other hand, by the very definition of $\Delta_k(t)$ we can calculate the same average as

$$\frac{1}{\tau}\sum_{t=1}^{\tau}\Delta_k(t) = \frac{\gamma_k(\tau+1)-\gamma_k(1)}{\tau}. \tag{55}$$

By setting the right hand's side of Eq. (54) equal to the right hand's side of Eq. (55), and taking the limit

$\tau \to \infty$, we obtain

$$\sum_{\omega} (p^k_{\tilde{\mathbf{a}}} - e^k_{\mathbf{a}}) \cdot v_\omega = 0. \tag{56}$$

where v_ω denotes the average abundance of observing $\omega = (\mathbf{a}, \tilde{\mathbf{a}})$ over time. Slightly rewriting this equation yields

$$\sum_{\omega} p^k_{\tilde{\mathbf{a}}} \cdot v_\omega = \sum_{\omega} e^k_{\mathbf{a}} \cdot v_\omega, \tag{57}$$

or equivalently,

$$\sum_{\tilde{\mathbf{a}}} p^k_{\tilde{\mathbf{a}}} \cdot \tilde{v}_{\tilde{\mathbf{a}}} = \sum_{\tilde{\mathbf{a}}} p^k_{\tilde{\mathbf{a}}} \sum_{\mathbf{a}} v_{(\mathbf{a},\tilde{\mathbf{a}})} = \sum_{\omega} p^k_{\tilde{\mathbf{a}}} \cdot v_\omega = \sum_{\omega} e^k_{\mathbf{a}} \cdot v_\omega = \sum_{\mathbf{a}} e^k_{\mathbf{a}} \sum_{\tilde{\mathbf{a}}} v_{(\mathbf{a},\tilde{\mathbf{a}})} = \sum_{\mathbf{a}} e^k_{\mathbf{a}} \cdot v_{\mathbf{a}} = \gamma_k, \tag{58}$$

which is Eq. (49). To obtain Eq. (50), we note that

$$1 - \gamma_k = 1 - \sum_{\tilde{\mathbf{a}}} \tilde{v}_{\tilde{\mathbf{a}}} \cdot p^k_{\tilde{\mathbf{a}}} = \sum_{\tilde{\mathbf{a}}} \tilde{v}_{\tilde{\mathbf{a}}} - \sum_{\tilde{\mathbf{a}}} \tilde{v}_{\tilde{\mathbf{a}}} \cdot p^k_{\tilde{\mathbf{a}}} = \sum_{\tilde{\mathbf{a}}} \tilde{v}_{\tilde{\mathbf{a}}} \cdot (1 - p^k_{\tilde{\mathbf{a}}}). \tag{59}$$

□

Based on Akin's Lemma, we are now able to describe which reactive strategies are partners when each of the games takes the form of a donation game with benefit b_k and cost c_k, respectively.

Proof of Proposition 1: Characterization of partner strategies in $\mathcal{R}_L$.
Let $\mathbf{p} = (p_{\mathbf{a}}) \in \mathcal{R}_L$ be a self-cooperative strategy (to which we refer as 'resident strategy'). In particular, it follows that the resident payoff (the resident strategy's payoff against itself) is given by

$$\pi = \sum_{k=1}^{m} b_k - c_k. \tag{60}$$

By deviating from the resident strategy, player 2 obtains a payoff that can be written as

$$\tilde{\pi} = \sum_{k=1}^{m} b_k \gamma_k - c_k \tilde{\gamma}_k, \tag{61}$$

where γ_k and $\tilde{\gamma}_k$ are the resulting average cooperation rates in a game between the resident and the deviating player. Strategy $\mathbf{p}$ is thus a Nash equilibrium if and only if

$$\sum_{k=1}^{m} b_k \gamma_k - c_k \tilde{\gamma}_k \leq \sum_{k=1}^{m} b_k - c_k, \tag{62}$$

which can be rewritten as

$$\sum_{k=1}^{m} b_k (1 - \gamma_k) - \sum_{k=1}^{m} c_k (1 - \tilde{\gamma}_k) \geq 0. \tag{63}$$

Due to Akin's Lemma and Eq. (48), the above inequality is equivalent to

$$\sum_{k=1}^{m} b_k \Big(\sum_{\tilde{\mathbf{a}}} \tilde{v}_{\tilde{\mathbf{a}}} \cdot (1 - p_{\tilde{\mathbf{a}}}^k) \Big) - \sum_{k=1}^{m} c_k \Big(\sum_{\tilde{\mathbf{a}}} \tilde{v}_{\tilde{\mathbf{a}}} (1 - e_{\tilde{\mathbf{a}}}^k) \Big) \geq 0. \tag{64}$$

Changing the order of summation, we obtain that $\mathbf{p}$ is a Nash equilibrium if and only if for all deviations

$$\sum_{\tilde{\mathbf{a}}} \tilde{v}_{\tilde{\mathbf{a}}} \cdot \left[\sum_{k=1}^{m} b_k (1 - p_{\tilde{\mathbf{a}}}^k) - c_k (1 - e_{\tilde{\mathbf{a}}}^k) \right] \geq 0. \tag{65}$$

In particular, this inequality needs to hold in case the deviating player uses a particular constant action profile $\tilde{\mathbf{a}}$ in every round, such that the resulting outcome distribution satisfies $v_{\tilde{\mathbf{a}}} = 1$. For that special case, it follows from (65) that any Nash equilibrium needs to satisfy

$$\sum_{k=1}^{m} b_k (1 - p_{\tilde{\mathbf{a}}}^k) - c_k (1 - e_{\tilde{\mathbf{a}}}^k) \geq 0, \tag{66}$$

for any action profile $\tilde{\mathbf{a}}$, which reproduces the condition for partners given in Eq. (23). Conversely, if condition (66) is satisfied for any $\tilde{\mathbf{a}}$, it follows that every summand on the left hand's side of Eq. (65) is non-negative. Hence also the sum is non-negative, and $\mathbf{p}$ is a Nash equilibrium. □

The proofs for defectors and semi-partners are similar.

Proof of Proposition 2: Characterization of defectors in $\mathcal{R}_L$.
Since the payoff of a self-defector is zero, the resident strategy $\mathbf{p}$ is a Nash equilibrium if and only if after any unilateral deviation the resident's cooperation rate γ_k and the deviating player's cooperation rate $\tilde{\gamma}_k$ satisfy

$$\sum_{k=1}^{m} b_k \gamma_k - c_k \tilde{\gamma}_k \leq 0. \tag{67}$$

By Akin's Lemma and Eq. (47), this condition is equivalent to

$$\sum_{k=1}^{m} b_k \Big(\sum_{\tilde{\mathbf{a}}} \tilde{v}_{\tilde{\mathbf{a}}} \cdot p_{\tilde{\mathbf{a}}}^k \Big) - c_k \Big(\sum_{\tilde{\mathbf{a}}} \tilde{v}_{\tilde{\mathbf{a}}} \cdot e_{\tilde{\mathbf{a}}}^k \Big) \leq 0. \tag{68}$$

Changing the order of summation yields

$$\sum_{\tilde{\mathbf{a}}} \tilde{v}_{\tilde{\mathbf{a}}} \cdot \left[\sum_{k=1}^{m} b_k \cdot p_{\tilde{\mathbf{a}}}^k - c_k \cdot e_{\tilde{\mathbf{a}}}^k \right] \leq 0. \tag{69}$$

Again, for $\mathbf{p}$ to be a Nash equilibrium, the above inequality needs to hold for all possible deviation strategies, and hence for the deviation strategy that unconditionally plays an arbitrary action profile $\tilde{\mathbf{a}}$ in

every round. It follows that

$$\sum_{k=1}^{m} b_k \cdot p^k_{\tilde{\mathbf{a}}} - c_k \cdot e^k_{\tilde{\mathbf{a}}} \le 0 \tag{70}$$

needs to hold for every action profile $\tilde{\mathbf{a}}$, which is condition (24) in Proposition 2. Conversely, if this condition is satisfied for all action profiles $\tilde{\mathbf{a}}$, then so is condition (69), and hence the self-defective strategy $\mathbf{p}$ is a Nash equilibrium. □

Proof of Proposition 3: Characterization of semi-partners in $\mathcal{R}_L$.
The self-payoff of a strategy that is self-cooperative in game k and self-defective in all other games is $b_k - c_k$. To be a Nash equilibrium, the resulting cooperation rates in a game against any deviation strategy need to satisfy

$$\sum_{l=1}^{m} b_l \gamma_l - c_l \tilde{\gamma}_l \le b_k - c_k, \tag{71}$$

or equivalently,

$$b_k(1-\gamma_k) - c_k(1-\tilde{\gamma}_k) - \sum_{l\neq k} b_l\gamma_l + c_l\tilde{\gamma}_l \ge 0. \tag{72}$$

By Akin's Lemma, and Eqs. (47) and (48), the previous inequality can be rewritten as

$$b_k\Big(\sum_{\tilde{\mathbf{a}}} \tilde{v}_{\tilde{\mathbf{a}}}(1-p^k_{\tilde{\mathbf{a}}})\Big) - c_k\Big(\sum_{\tilde{\mathbf{a}}} \tilde{v}_{\tilde{\mathbf{a}}}(1-e^k_{\tilde{\mathbf{a}}})\Big) - \sum_{l\neq k} b_l\Big(\sum_{\tilde{\mathbf{a}}} \tilde{v}_{\tilde{\mathbf{a}}} \cdot p^k_{\tilde{\mathbf{a}}}\Big) + c_l\Big(\sum_{\tilde{\mathbf{a}}} \tilde{v}_{\tilde{\mathbf{a}}} \cdot e^l_{\tilde{\mathbf{a}}}\Big) \ge 0. \tag{73}$$

Reversing the order of summation then yields

$$\sum_{\tilde{\mathbf{a}}} \tilde{v}_{\tilde{\mathbf{a}}} \left[b_k(1-p^k_{\tilde{\mathbf{a}}}) - c_k(1-e^k_{\tilde{\mathbf{a}}}) - \sum_{l\neq k} b_l \cdot p^k_{\tilde{\mathbf{a}}} + c_l \cdot e^l_{\tilde{\mathbf{a}}} \right] \ge 0. \tag{74}$$

In particular, this inequality needs to hold when the co-player's action profile is given by the same tuple $\tilde{\mathbf{a}}$ in each round. Thus $\mathbf{p}$ can only be a Nash equilibrium if for all action profiles $\tilde{\mathbf{a}}$,

$$b_k(1-p^k_{\tilde{\mathbf{a}}}) - c_k(1-e^k_{\tilde{\mathbf{a}}}) - \sum_{l\neq k} b_l \cdot p^k_{\tilde{\mathbf{a}}} + c_l \cdot e^l_{\tilde{\mathbf{a}}} \ge 0. \tag{75}$$

This is condition (25) in Proposition 3. Conversely, given that inequality (75) holds for any action profile $\tilde{\mathbf{a}}$, it also holds for the linear combination in Eq. (74). Hence $\mathbf{p}$ is a Nash equilibrium. □

Proof of Proposition 4: Partners, defectors, and semi-partners in $\mathcal{R}_U$.

1. For a strategy $\mathbf{p} = (p^1_{a_1}, \ldots, p^m_{a_m}) \in \mathcal{R}_U$ to be self-cooperative, it follows that $p^k_C = 1$ for all k.

   To show that $p^k_D \le 1 - c^k/b^k$ is necessary for the strategy to be a Nash equilibrium, assume to the contrary that there is some game l for which the inequality is not satisfied. Consider the possible

deviation strategy $\tilde{\mathbf{p}}$ defined by

$$\tilde{p}^k_{a_k} = \begin{cases} 0 & \text{if } k=l \\ p^k_{a_k} & \text{otherwise} \end{cases} \tag{76}$$

That is, $\tilde{\mathbf{p}}$ is identical to $\mathbf{p}$ except for game l in which it plays *ALLD*. Because games are unlinked, it follows that $\tilde{\mathbf{p}}$ obtains the same payoff as $\mathbf{p}$ in every game except for game l. In game l its payoff is $p^l_D \cdot b_l > b_l - c_l$. Hence the deviation payoff exceeds the mutual cooperation payoff that a $\mathbf{p}$-player gets against itself.

Conversely, to show that $p^k_D \leq 1 - c^k/b^k$ is sufficient for the strategy to be a Nash equilibrium, we note that the strategy's representation in the linked strategy space satisfies all conditions in (23). It follows that $\mathbf{p}$ is a Nash equilibrium within the linked strategy space, and therefore also in the unlinked space.

2. For the strategy to be self-defective, it follows that $p^k_D = 0$ for all games k.

   To show that $p^k_C \leq c_k/b_k$ is necessary, again suppose there is a game l for which the inequality is not satisfied, and consider the deviation strategy $\tilde{\mathbf{p}}$ defined by

$$\tilde{p}^k_{a_k} = \begin{cases} 1 & \text{if } k=l \\ p^k_{a_k} & \text{otherwise} \end{cases} \tag{77}$$

   This strategy coincides with $\mathbf{p}$ in all games except game l, where it plays *ALLC*. The payoff of $\tilde{\mathbf{p}}$ against $\mathbf{p}$ in game l is $b_l\, p^l_C - c_l > 0$, which exceeds the payoff $\mathbf{p}$ gets against itself.

   Conversely, if $\mathbf{p} \in \mathcal{R}_U$ satisfies $p^k_D = 0$ and $p^k_C \leq c_k/b_k$ for all k, it is straighforward to check that the strategy's representation in $\mathcal{R}_L$ satisfies all conditions in (24).

3. For game-k semi-partners, the proof is analogous to the previous two cases. If $p^k_D > 1 - c_k/b_k$, it pays to deviate by always defecting in game k. If there is some $l \neq k$ for which $p^l_C > c_l/b_l$, it pays to deviate by always cooperating in game l. The converse direction is again by showing that the linked representation of the strategy satisfies the conditions in (25). □

Proof of Proposition 5: Stability of WSLS *and* CIC.
To derive the equilibrium conditions for *WSLS* and *CIC* in a multichannel game without discounting, we prove a slightly more general result, for a multichannel game with discounting. To this end, we assume that players discount game payoffs in round t by a factor of δ^t, where $0 < \delta < 1$. A player's repeated game payoff in game k is then defined as the weighted sum

$$\pi^k_i = (1-\delta) \sum_{t=1}^{\tau} \delta^t \pi^k_i(t). \tag{78}$$

Importantly, for $\delta < 1$ the outcome of the very first round now affects the players' payoffs. In line with our notion of self-cooperativeness, we assume that a player adopting either *WSLS* or *CIC* cooperates in the initial round (when no previous-round history is available). In the following, we show that under these conditions, *WSLS* is a subgame perfect equilibrium if and only if

$$\delta \geq \frac{c_k}{b_k - c_k} \quad \text{for all games } k. \tag{79}$$

Similarly, we show *CIC* is a subgame perfect equilibrium if and only if

$$\delta \geq \frac{\sum_{k=1}^{m} c_k}{\sum_{k=1}^{m} b_k - c_k}. \tag{80}$$

Proposition 5 then follows from Eqs. (79) and (80) by taking the limit $\delta \to 1$. Conditions (79) and (80) are derived as follows.

1. *Stability of* WSLS. To prove condition (79), we use the one-shot deviation principle[66]. That is, we show that there is no history after which it pays to deviate for a single round and then to act as prescribed by *WSLS* in all subsequent rounds. To this end, consider a game between a mutant and a resident *WSLS* player, and suppose the history is such that the resident chooses some given action profile $\mathbf{a} = (a_1, \ldots, a_m)$ in the next round. For this action profile, define the index sets

$$I_C = \Big\{ k \in \{1, \ldots, m\} \;\Big|\; a_k = C \Big\} \quad \text{and} \quad I_D = \Big\{ k \in \{1, \ldots, m\} \;\Big|\; a_k = D \Big\}. \tag{81}$$

That is, the set I_C comprises all games in which the resident *WSLS* player would cooperate, whereas I_D comprises the games in which the resident would defect. Suppose the mutant deviates for one round, by choosing the action profile $\tilde{\mathbf{a}} = (\tilde{a}_1, \ldots, \tilde{a}_m)$. We define the index sets

$$L_C = \Big\{ k \in I_C \;\Big|\; \tilde{a}_k = D \Big\} \quad \text{and} \quad L_D = \Big\{ k \in I_D \;\Big|\; \tilde{a}_k = C \Big\}. \tag{82}$$

These sets L_C and L_D thus correspond to the games in which the mutant deviates. If the mutant chooses to adopt *WSLS*, her action profile is $\tilde{\mathbf{a}} = \mathbf{a}$ and her continuation payoff becomes

$$\pi_W = \sum_{k \in I_C} (b_k - c_k) + \delta \sum_{k \in I_D} (b_k - c_k). \tag{83}$$

This equation reflects that for games in I_C, two *WSLS* players would cooperate indefinitely; for games in I_D they would defect for one round and then cooperate indefinitely. If the mutant instead deviates for one round and chooses $\tilde{\mathbf{a}} \neq \mathbf{a}$, her continuation payoff becomes

$$\pi_M = \sum_{k \in I_C \setminus L_C} (b_k - c_k) + \sum_{k \in L_C} \big((1-\delta) b_k + \delta^2 (b_k - c_k)\big) + \delta \sum_{k \in I_D \setminus L_D} (b_k - c_k) + \sum_{k \in L_D} \big(-(1-\delta) c_k + \delta^2 (b_k - c_k)\big) \tag{84}$$

Such a one-shot deviation is unprofitable if and only if $\pi_W \geq \pi_M$, that is if and only if

$$-\sum_{k\in L_C} c_k \;+\; \delta\sum_{k\in L_C}(b_k - c_k) + \sum_{k\in L_D} c_k \;+\; \delta\sum_{k\in L_D}(b_k - c_k) \;\geq\; 0. \tag{85}$$

In particular, this condition needs to hold if $L_D = \emptyset$ and $L_C = \{k\}$ for any game k, which yields condition (79). Conversely, if condition (79) is satisfied for all k, then the sum of the first two terms in (85) are non-negative, and therefore so is the entire sum.

2. *Stability of* CIC. For the proof we again use the one-shot deviation principle. To this end, consider a resident player with strategy *CIC*, and suppose first the history is such that the *CIC* player would cooperate in the next round (in all games). When adopting the same strategy, the mutant's continuation payoff is

$$\pi_C = \sum_k^m b_k - c_k. \tag{86}$$

If she deviates instead, it follows by the definition of *CIC* that the mutant should deviate in all m games simultaneously to maximize her continuation payoff, which yields

$$\pi_M = (1-\delta)\sum_k^m b_k \;+\; \delta^2\sum_k^m b_k - c_k. \tag{87}$$

The condition $\pi_C \geq \pi_M$ is equivalent to condition (80).
Now suppose the history is such that the *CIC* resident would defect in the next round. Then by playing the same strategy the mutant obtains the continuation payoff

$$\pi_C = \delta\sum_k^m b_k - c_k. \tag{88}$$

If the mutant instead deviates from the resident strategy, the mutant pays the respective cooperation cost; in addition, the resident will also defect in the subsequent round. Even if we assume that the cooperation cost is negligible, the mutant's continuation payoff will satisfy

$$\pi_M < \delta^2\sum_k^m b_k - c_k, \tag{89}$$

which is always lower than π_C. Hence, such a deviation is always unprofitable. □

We note that when the conditions (79) and (80) are met strictly, then the two strategies are what Boyd[35] calls 'strong perfect equilibria'. As shown there, such strategies are not only Nash equilibria, but they are evolutionarily stable if the game is subject to rare errors. Intuitively, errors make sure that every possible history is reached with positive probability. The strong equilibrium property then makes sure that the respective strategy is a strict best response to itself after any history.

MATLAB code used for the numerical results

In the following, we provide the MATLAB code that we have used to derive all our numerical results. The first routine describes the game dynamics of a multichannel game. It takes the players' reactive strategies and the game parameters as input, and computes the players' payoffs and their cooperation rates as output, as described in Section "Calculation of payoffs". The second routine describes the evolutionary dynamics. It simulates the pairwise comparison process in the limit of rare mutations, as described in Section "Evolutionary dynamics".

Game dynamics

```
function [pi,coop,v,W,A]=GameDynamics(p1,p2,u);

% [pi,coop]=GameDynamics(p1,p2,u);
% Computes payoffs when two players engage in m games
% pi=[pi1,pi2] ..  Payoffs of the two players
% coop=[cp11,cp12; ..  ; cpm1,cpm2] ..  Cooperation rates across the m games
% v ..  Invariant distribution of game dynamics
% W ..  Transition matrix
% A ..  List of possible action profiles
% p1,p2 ..  m x 2^m matrices for reactive strategies of the two players
% u=[R1,S1,T1,P1; ..  ; Rm,Sm,TmP,Pm] ..  Payoffs


%% PREPARATIONS %%
m=size(u,1); nA=2^m; % Number of possible action profiles for each player
pi=zeros(1,2); coop=zeros(m,2); % Initiating the output variables pi, coop
A=zeros(nA,m); % List of possible action profiles; 1=C, 0=D
for i=1:nA
  for j=1:m
    A(i,j)=mod(floor((i-1)/2^(m-j)),2);
  end
end
A=A(nA:-1:1,:); % Reverse the order of action profiles from ALLC to ALLD


%% CONSTRUCTION OF THE TRANSITION MATRIX W %%
W=zeros(nA^2,nA^2);
for i1=1:nA % Previous action profile of player 1
  for i2=1:nA % Previous action profile of player 2
    P1=p1(:,i2); P2=p2(:,i1); % Respective conditional strategies of the two players
    for i1N=1:nA % Next action profile of player 1
      for i2N=1:nA % Next action profile of player 2
        w=1;
        for k=1:m
          if A(i1N,k)==1 % Player 1 cooperates in next round of game k
            w=w*P1(k);
          else % Player 1 defects in next round
            w=w*(1-P1(k));
          end
          if A(i2N,k)==1 % Player 2 cooperates in next round of game k
```

```
      w=w*P2(k);
     else% Player 2 defects in next round
      w=w*(1-P2(k));
     end
    end
    W((i1-1)*nA+i2,(i1N-1)*nA+i2N)=w; % Storing transition probability in matrix
   end
  end
 end
end
v=null(W'-eye(nA^2,nA^2)); v=v/sum(v); % Calculating the invariant distribution

%% CONSTRUCTION OF THE LIST OF PAYOFFS AND ACTIONS %%
Pay=zeros(nA^2,2); % List of possible one-shot payoffs
A1=zeros(nA^2,m); A2=A1; % List of both players' actions
for i1=1:nA % Action profile of player 1
 for i2=1:nA % Action profile of player 2
  A1((i1-1)*nA+i2,:)=A(i1,:);
  A2((i1-1)*nA+i2,:)=A(i2,:);
  piA=[0,0];
  for k=1:m
   a1=A(i1,k); a2=A(i2,k);
   if a1==1 & a2==1
    piA=piA+[u(k,1),u(k,1)];
   elseif a1==1 & a2==0
    piA=piA+[u(k,2),u(k,3)];
   elseif a1==0 & a2==1
    piA=piA+[u(k,3),u(k,2)];
   elseif a1==0 & a2==0
    piA=piA+[u(k,4),u(k,4)];
   end
  end
  Pay((i1-1)*nA+i2,:)=piA;
 end
end

%% COMPUTING PAYOFFS AND COOPERATION RATES %%
pi=Pay'*v; cp1=A1'*v; cp2=A2'*v; coop=[cp1'; cp2']';
end
```

Evolutionary dynamics

```
function [Pi,Coop,SEnd,u,Data]=EvolutionaryDynamics(u,N,s,nGen,link);

% [Pi,Coop1,Coop2,SEnd,Data]=EvolutionaryDynamics(u,N,s,nGen,link)
% Simulates the rare-mutation pairwise imitation dynamics for nGen time steps,
% in a population of size N with selection strength s
% u=[R1,S1,T1,P1; ..  ; Rm,Sm,TmP,Pm] ..  Payoffs
% link == 1 ..  linked strategy space, link==0 ..  unlinked strategy space
% Pi ..  Payoff vector over time
% Coop ..  Cooperation matrix across all games
```

```
% SEnd ..  Final strategy
% Data ..  String that gives a protocol of the used parameters

C=clock; rng(C(5)*60+C(6)); % Setting up the random number generator

%% PARAMETERS AND PREPARATIONS %%
m=size(u,1); % Number of games
p=zeros(m,2^m); % Initially, population applies ALLD
[pi,cp]=GameDynamics(p,p,u); % Calculate initial payoffs and cooperation rates
Pi=zeros(1,nGen); pi=pi(1); Pi(1)=pi; % Initializing the output
Coop=zeros(m,nGen); coop=cp(:,1); Coop(:,1)=coop;
Data=['N=',num2str(N),'; s=',num2str(s),'; nGen=',num2str(nGen)];


%% CREATING A LIST OF POSSIBLE ACTION PROFILES %%
nA=2^m; % Number of possible action profiles for each player
A=zeros(nA,m); % List of possible action profiles; 1 ..  C, 0 ..  D
for i=1:nA
 for j=1:m
  A(i,j)=mod(floor((i-1)/2^(m-j)),2);
 end
end
A=A(nA:-1:1,:); % Reverse the order of action profiles from ALLC to ALLD


%% EVOLUTIONARY PROCESS %%
for i=2:nGen
 % Step 1:  Introducing a mutant
 if link==1 Mut=rand(m,2^m); % Linked mutant
 elseif link==0
  Mut=CreateUnlinkedMutant(m,A); % Unlinked mutant
 end

 % Step 2:  Determining whether mutant fixes in the population
 [PayM,coopM]=createPayM(p,Mut,u,pi); % Calculate mutant's payoff
 Rho=CalcRho(PayM,N,s); % Calculate mutant's fixation probability
 if Rho>rand(1) % If mutant fixes
  p=Mut; pi=PayM(2,2); coop=coopM; % Updating the running variables
 end

 % Storing the current resident values in the output vectors
 Pi(i)=pi; Coop(:,i)=coop;
end
SEnd=p;
end


function Rho=CalcRho(PayM,N,s);
% Subroutine to compute fixation probability of a mutant (1) in resident (2)
pi1=[N-1:-1:1; 0:N-2]'*PayM(2,:)'/(N-1); % Mutant payoffs
pi2=[N-2:-1:0; 1:N-1]'*PayM(1,:)'/(N-1); % Resident payoffs
Rho=1/(sum(cumprod(exp(-s*(pi1-pi2))))+1); % Fixation probability
end
```

```
function [PayM,coopM]=createPayM(Res,Mut,u,pi)
% Subroutine that creates 2x2 payoff matrix between mutant and resident
piRM=GameDynamics(Res,Mut,u);
[piMM,coopM]=GameDynamics(Mut,Mut,u);
PayM=[pi, piRM(1); piRM(2), piMM(1)];
coopM=coopM(:,1);
end
```

```
function Mut=CreateUnlinkedMutant(m,A);
% Subroutine that creates mutant strategies in unlinked strategy space
Mut=zeros(m,2^m);
for k=1:m
 iC=find(A(:,k)==1); % Find action profiles with co-player's C in game k
 Mut(k,iC)=rand(1); % Assign same cooperation probability to all these profiles
 iD=find(A(:,k)==0); % Similar for co-players who played D in game k
 Mut(k,iD)=rand(1);
end
end
```

Supplementary References

[1] Cressman, R., Gaunersdorfer, A. & Wen, J. F. Evolutionary and dynamic stability in symmetric evolutionary games with two independent decisions. *International Game Theory Review* **2** (2000).

[2] Chamberland, M. & Cressman, R. An example of dynamic (in)consistency in symmetric extensive form evolutionary games. *Games and Economic Behavior* **30**, 319–326 (2000).

[3] Hashimoto, K. Unpredictability induced by unfocused games in evolutionary game dynamics. *Journal of Theoretical Biology* **241**, 669–675 (2006).

[4] Venkateswaran, V. R. & Gokhale, C. S. Evolutionary dynamics of complex multiple games. *Proceedings of the Royal Society B* **286**, 20190900 (2019).

[5] Bernheim, D. & Whinston, M. D. Multimarket contact and collusive behavior. *The RAND journal of economics* **21**, 1–26 (1990).

[6] Matsushima, H. Multimarket contact, imperfect monitoring, and implicit collusion. *Journal of Economic Theory* **98**, 158–178 (2001).

[7] Jayachandran, S., Gimeno, J. & R., V. P. The theory of multimarket competition: A synthesis and implications for marketing strategy. *Journal of Marketing* **63**, 49–66 (1999).

[8] Fehr, E. & Gächter, S. Altruistic punishment in humans. *Nature* **415**, 137–140 (2002).

[9] Dreber, A., Rand, D. G., Fudenberg, D. & Nowak, M. A. Winners don't punish. *Nature* **452**, 348–351 (2008).

[10] Gächter, S., Renner, E. & Sefton, M. The long-run benefits of punishment. *Science* **322**, 1510 (2008).

[11] Herrmann, B., Thöni, C. & Gächter, S. Antisocial punishment across societies. *Science* **319**, 1362–1367 (2008).

[12] Rand, D. G., Dreber, A., Ellingsen, T., Fudenberg, D. & Nowak, M. A. Positive interactions promote public cooperation. *Science* **325**, 1272–1275 (2009).

[13] Hilbe, C., Traulsen, A., Röhl, T. & Milinski, M. Democratic decisions establish stable authorities that overcome the paradox of second-order punishment. *Proceedings of the National Academy of Sciences USA* **111**, 752–756 (2014).

[14] Milinski, M., Semmann, D. & Krambeck, H. J. Reputation helps solve the 'tragedy of the commons'. *Nature* **415**, 424–426 (2002).

[15] Hauser, O. P., Hendriks, A., Rand, D. G. & Nowak, M. A. Think global, act local: Preserving the global commons. *Scientific Reports* **6**, 36079 (2016).

[16] Sigmund, K., Hauert, C. & Nowak, M. A. Reward and punishment. *Proceedings of the National Academy of Sciences USA* **98**, 10757–10762 (2001).

[17] Fowler, J. H. Altruistic punishment and the origin of cooperation. *Proceedings of the National Academy of Sciences USA* **102**, 7047–7049 (2005).

[18] Ohtsuki, H., Iwasa, Y. & Nowak, M. A. Indirect reciprocity provides only a narrow margin of efficiency for costly punishment. *Nature* **457**, 79–82 (2009).

[19] Szolnoki, A. & Perc, M. Reward and cooperation in the spatial public goods game. *Europhysics Letters* **92**, 38003 (2010).

[20] Helbing, D., Szolnoki, A., Perc, M. & Szabo, G. Punish, but not too hard: how costly punishment spreads in the spatial public goods game. *New Journal of Physics* **12**, 083005 (2010).

[21] Hilbe, C. & Sigmund, K. Incentives and opportunism: from the carrot to the stick. *Proceedings of the Royal Society B* **277**, 2427–2433 (2010).

[22] Cressman, R., Song, J.-W., Zhang, B.-Y. & Tao, Y. Cooperation and evolutionary dynamics in the public goods game with instutional incentives. *Journal of Theoretical Biology* **299**, 144–151 (2012).

[23] Sasaki, T., Brännström, Å., Dieckmann, U. & Sigmund, K. The take-it-or-leave-it option allows small penalties to overcome social dilemmas. *Proceedings of the National Academy of Sciences USA* **109**, 1165–1169 (2012).

[24] Okada, I., Yamamoto, H., Toriumi, F. & Sasaki, T. The effect of incentives and meta-incentives on the evolution of cooperation. *PLoS Computational Biology* **11**, e1004232 (2015).

[25] Nikiforakis, N. Punishment and counter-punishment in public good games: Can we really govern ourselves? *Journal of Public Economics* **92**, 91–112 (2008).

[26] Szolnoki, A. & Perc, M. Antisocial pool rewarding does not deter public cooperation. *Proceedings of the Royal Society B* **282**, 20151975 (2015).

[27] dos Santos, M. & Peña, J. Antisocial rewarding in structured populations. *Scientific reports* **7**, 6212 (2017).

[28] Rapoport, A. & Chammah, A. M. *Prisoner's Dilemma* (University of Michigan Press, Ann Arbor, 1965).

[29] Kerr, B., Godfrey-Smith, P. & Feldman, M. W. What is altruism? *Trends in Ecology & Evolution*

19, 135–140 (2004).

[30] Nowak, M. A. Evolving cooperation. *Journal of Theoretical Biology* **299**, 1–8 (2012).

[31] Sigmund, K. *The Calculus of Selfishness* (Princeton Univ. Press, Princeton, NJ, 2010).

[32] Boyd, R. & Lorberbaum, J. No pure strategy is evolutionary stable in the iterated prisoner's dilemma game. *Nature* **327**, 58–59 (1987).

[33] García, J. & van Veelen, M. In and out of equilibrium I: Evolution of strategies in repeated games with discounting. *Journal of Economic Theory* **161**, 161–189 (2016).

[34] García, J. & van Veelen, M. No strategy can win in the repeated prisoner's dilemma: Linking game theory and computer simulations. *Frontiers in Robotics and AI* **5**, 102 (2018).

[35] Boyd, R. Mistakes allow evolutionary stability in the repeated Prisoner's Dilemma game. *Journal of Theoretical Biology* **136**, 47–56 (1989).

[36] Mailath, G. J. & Samuelson, L. *Repeated games and reputations* (Oxford Univ. Press, Oxford, UK, 2006).

[37] Hilbe, C., Chatterjee, K. & Nowak, M. A. Partners and rivals in direct reciprocity. *Nature Human Behaviour* **2**, 469–477 (2018).

[38] Nowak, M. A. & Sigmund, K. The evolution of stochastic strategies in the prisoner's dilemma. *Acta Applicandae Mathematicae* **20**, 247–265 (1990).

[39] Nowak, M. A. & Sigmund, K. Tit for tat in heterogeneous populations. *Nature* **355**, 250–253 (1992).

[40] Press, W. H. & Dyson, F. D. Iterated prisoner's dilemma contains strategies that dominate any evolutionary opponent. *PNAS* **109**, 10409–10413 (2012).

[41] Traulsen, A., Nowak, M. A. & Pacheco, J. M. Stochastic dynamics of invasion and fixation. *Physical Review E* **74**, 011909 (2006).

[42] Traulsen, A., Pacheco, J. M. & Nowak, M. A. Pairwise comparison and selection temperature in evolutionary game dynamics. *Journal of Theoretical Biology* **246**, 522–529 (2007).

[43] Szabó, G. & Tőke, C. Evolutionary Prisoner's Dilemma game on a square lattice. *Physical Review E* **58**, 69–73 (1998).

[44] Blume, L. E. The statistical mechanics of strategic interaction. *Games and Economic Behavior* **5**, 387–424 (1993).

[45] Fudenberg, D. & Imhof, L. A. Imitation processes with small mutations. *Journal of Economic Theory* **131**, 251–262 (2006).

[46] Imhof, L. A. & Nowak, M. A. Stochastic evolutionary dynamics of direct reciprocity. *Proceedings of the Royal Society B* **277**, 463–468 (2010).

[47] McAvoy, A. Comment on "Imitation processes with small mutations". *J. Econ. Theory* **159**, 66–69 (2015).

[48] Wu, B., Gokhale, C. S., Wang, L. & Traulsen, A. How small are small mutation rates? *Journal of Mathematical Biology* **64**, 803–827 (2012).

[49] Nowak, M. A., Sasaki, A., Taylor, C. & Fudenberg, D. Emergence of cooperation and evolutionary

stability in finite populations. *Nature* **428**, 646–650 (2004).

[50] Traulsen, A. & Hauert, C. Stochastic evolutionary game dynamics. In Schuster, H. G. (ed.) *Reviews of Nonlinear Dynamics and Complexity*, 25–61 (Wiley-VCH, Weinheim, 2009).

[51] Hilbe, C., Traulsen, A. & Sigmund, K. Partners or rivals? Strategies for the iterated prisoner's dilemma. *Games and Economic Behavior* **92**, 41–52 (2015).

[52] Akin, E. The iterated prisoner's dilemma: Good strategies and their dynamics. In Assani, I. (ed.) *Ergodic Theory, Advances in Dynamics*, 77–107 (de Gruyter, Berlin, 2016).

[53] Akin, E. What you gotta know to play good in the iterated prisoner's dilemma. *Games* **6**, 175–190 (2015).

[54] Stewart, A. J. & Plotkin, J. B. From extortion to generosity, evolution in the iterated prisoner's dilemma. *Proceedings of the National Academy of Sciences USA* **110**, 15348–15353 (2013).

[55] Stewart, A. J. & Plotkin, J. B. Collapse of cooperation in evolving games. *Proceedings of the National Academy of Sciences USA* **111**, 17558 – 17563 (2014).

[56] Stewart, A. J. & Plotkin, J. B. The evolvability of cooperation under local and non-local mutations. *Games* **6**, 231–250 (2015).

[57] Stewart, A. J. & Plotkin, J. B. Small groups and long memories promote cooperation. *Scientific Reports* **6**, 26889 (2016).

[58] Molander, P. The optimal level of generosity in a selfish, uncertain environment. *Journal of Conflict Resolution* **29**, 611–618 (1985).

[59] Nowak, M. A. & Sigmund, K. A strategy of win-stay, lose-shift that outperforms tit-for-tat in the Prisoner's Dilemma game. *Nature* **364**, 56–58 (1993).

[60] Kraines, D. P. & Kraines, V. Y. Evolution of learning among pavlov strategies in a competitive environment with noise. *Journal of Conflict Resolution* **39**, 439–466 (1995).

[61] Hauert, C. & Schuster, H. G. Effects of increasing the number of players and memory size in the iterated prisoner's dilemma: a numerical approach. *Proceedings of the Royal Society B* **264**, 513–519 (1997).

[62] Baek, S. K., Jeong, H. C., Hilbe, C. & Nowak, M. A. Comparing reactive and memory-one strategies of direct reciprocity. *Scientific Reports* **6**, 25676 (2016).

[63] Pinheiro, F. L., Vasconcelos, V. V., Santos, F. C. & Pacheco, J. M. Evolution of all-or-none strategies in repeated public goods dilemmas. *PLoS Comput Biol* **10**, e1003945 (2014).

[64] Hilbe, C., Martinez-Vaquero, L. A., Chatterjee, K. & Nowak, M. A. Memory-n strategies of direct reciprocity. *Proceedings of the National Academy of Sciences USA* **114**, 4715–4720 (2017).

[65] Selten, R. Spieltheoretische Behandlung eines Oligopolmodells mit Nachfrageträgheit. *Zeitschrift für die gesamte Staatswissenschaft* **121**, 301–324 (1965).

[66] Fudenberg, D. & Tirole, J. *Game Theory* (MIT Press, Cambridge, 1998), 6th edn.

[67] van Veelen, M., García, J., Rand, D. G. & Nowak, M. A. Direct reciprocity in structured populations. *Proceedings of the National Academy of Sciences USA* **109**, 9929–9934 (2012).

[68] Taylor, C. & Nowak, M. A. Transforming the dilemma. *Evolution* **61**, 2281–2292 (2007).

[69] Hauert, C. & Doebeli, M. Spatial structure often inhibits the evolution of cooperation in the snowdrift game. *Nature* **428**, 643–646 (2004).

[70] Doebeli, M. & Hauert, C. Models of cooperation based on the prisoner's dilemma and the snowdrift game. *Ecology Letters* **8**, 748–766 (2005).

[71] Diekmann, A. Volunteer's dilemma. *Journal of Conflict Resolution* **29**, 605–610 (1985).

[72] Iyer, S. & Killingback, T. Evolution of cooperation in social dilemmas on complex networks. *PLoS Computational Biology* **12**, e1004779 (2016).
